# Acidosis Regulates Microtubule Dynamics via the β1 Integrin/RhoA/CRMP‑2 Axis

**DOI:** 10.1021/jacs.5c20041

**Published:** 2026-05-20

**Authors:** Dariusz Lachowski, Ernesto Cortes, Vasyl Mykuliak, Miguel Fernandez-de la Torre, Ander Bastida Urkiza, Arrate Muñoz-Barrutia, Daniel Garcia-Gonzalez, Vesa Hytönen, Armando del Rio Hernandez

**Affiliations:** † Department of Neuroscience and Biomedical Sciences, 16726 Universidad Carlos III de Madrid, Getafe, Madrid 28903, Spain; ‡ Department of Bioengineering, 4615 Imperial College London, London SW7 2AZ, U.K.; § Department of Physiology, School of Medicine, 16722 Autonomous University of Madrid, Madrid 28029, Spain; ∥ Faculty of Medicine and Health Technology, 7840 Tampere University, Tampere 33100, Finland; ⊥ Fimlab Laboratories, Tampere 33013, Finland; # Department of Continuum Mechanics and Structural Analysis, Universidad Carlos III de Madrid, Leganés, Madrid 28911, Spain; ∇ 60Nd S.L., Avda. Gregorio Peces Barba 1, Leganés, Madrid 28919, Spain

## Abstract

Microtubules are dynamic cytoskeletal filaments that have important organizing roles in all eukaryotic cells. Early *in vitro* studies on the effect of pH on microtubule stability focused on microtubules isolated from whole cell lysates, which can only replicate changes in intracellular pH. However, how extracellular pH can affect microtubule dynamics remains unclear. Here, we report that acidosis activates β1 integrin by increasing its affinity for RGD-containing ligands through the displacement of divalent ions in the metal-ion-binding sites of β1 integrin extracellular domain via protonation of Asp138. This induces the activation of RhoA and its downstream effector ROCK, which, via phosphorylation of Collapsin Response Mediator Protein-2 (CRMP-2), negatively regulates microtubules stability and changes the positioning and architecture of the Golgi apparatus. Thereby, extracellular pH modulates microtubule dynamics, which could have important consequences for intracellular organization, cell polarization, vesicular trafficking, nucleocytoplasmic shuttling, and cell division. There are multiple processes in human physiology associated with acidosis. The presented mechanochemical mechanism that links low extracellular pH and microtubule stability may serve as a blueprint for advancing our knowledge of cellular transport and exploring potential targets for drug development.

## Graphical abstract

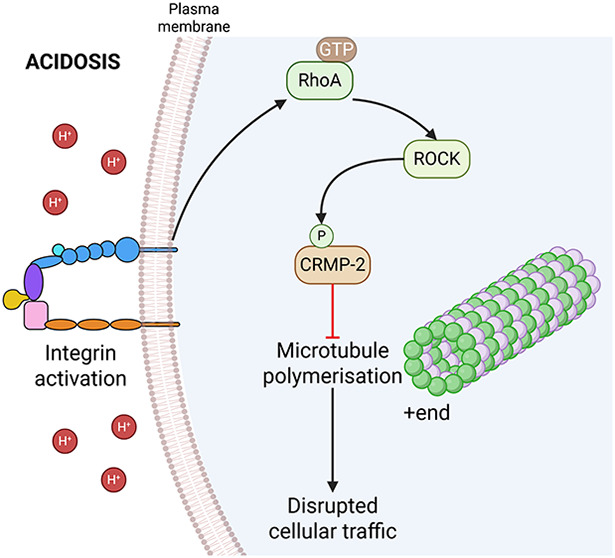

## 1 Introduction

Microtubules (MTs) are highly dynamic tubular protein structures that constitute one of the core components of the cytoskeleton. The structure of microtubules is polarized, with a fast growing (+) end that extends toward the cell periphery and a more static (−) end that associates with the microtubule-organizing center near the nucleus or exists as a free (−) end.^1,2^ The polarization of the microtubule network is central to its function as the organizational framework of the cells: it establishes cell directionality and directs intracellular transport, determines the localization and distribution of organelles, and forms the mitotic spindle that orchestrates cell division. MTs also contribute to cell morphology, mechanosensing, and locomotion through their interaction with the actomyosin cytoskeleton via spectraplakins and GAS2-like proteins,^3^ and coordinate actomyosin contractility by sequestering Rho GEFs.^4^ The ability of MTs to coordinate cell behavior in response to microenvironmental stimuli relies on their dynamic instability, that is, the ability to rapidly switch from growth to disassembly (catastrophe) and vice versa (rescue), which enables rapid reorganization of the MT network. Microtubule stability and dynamics at the (+) end are governed by the interplay between a wide array of microtubule-associated proteins (MAPs). They are key regulators of microtubule stability and organization, linking dynamic polymer networks to diverse cellular processes. MAPs promote microtubule assembly, prevent depolymerization, and facilitate interactions with other cytoskeletal elements, membranes, and signaling complexes. By modulating microtubule dynamics, MAPs coordinate essential functions, such as intracellular transport, cell migration, and mitotic spindle formation. Importantly, certain MAPs also act as downstream effectors of signaling pathways, enabling extracellular cues to directly influence the cytoskeletal behavior and cellular responses. MAPs include (+) end-tracking proteins (+TIPs), which stabilize microtubules, and microtubule disassembling proteins such as kinesin-13s.^2,5^

Early *in vitro* studies into the effect of pH on microtubule stability focused on microtubules isolated from whole cell lysates, which can only replicate intracellular changes in pH.^6,7^ However, eukaryotic cells use a system of ion transport mechanisms to keep their intracellular pH (pHi) constant (around 7.2), which is crucial for cellular events including proliferation.^8^ Cancer cells maintain a slightly alkaline intracellular pH thanks to a variety of effective acid extrusion mechanisms, including passive combined H^+^ and lactate monocarboxylate transporters (MCTs) and active H^+^/K^+^ and V-type ATPase pumps, among others.^9–11^ This resistance to changes in pHi suggests that acidosis-mediated MT modulation may not be dependent on the direct effect of pH on tubulin but instead is mediated by acid-sensing surface receptors that trigger intracellular signaling in response to extracellular pH (pHe).

The physiological extracellular pH of human tissues is tightly maintained within a narrow, slightly alkaline range of 7.35 to 7.45.^12^ This stability is crucial for normal cellular function, including enzyme activity, protein folding, and maintenance of the proper charge of biomolecules. In contrast, many pathological conditions, such as chronic inflammation, ischemia, and solid tumors, are characterized by a pronounced drop in the pHe, a state known as extracellular acidosis. For instance, the extracellular microenvironment of tumors is a hallmark of malignancy, with pHe values of 6.4–7.0 due to the high rate of aerobic glycolysis (the Warburg effect) and poor vascularization.^13^ In highly aggressive tumors, such as breast cancer and pancreatic ductal adenocarcinoma (PDAC), the pH can fall even lower. This acidic environment promotes tumor progression by increasing invasiveness, metastasis, and resistance to therapies, while also suppressing the antitumor immune response.^14^

Integrins are obligate heterodimeric transmembrane proteins involved in the adhesion and mechanical communication between cells and the extracellular matrix (ECM).^15^ Upon engagement, the headpiece of the integrin dimer ectodomain binds to ECM fibers, while its intracellular tail connects to the actomyosin cytoskeleton through a network of adaptor and linker proteins that includes talin, paxillin, and vinculin among others.^16^ Integrins play a well-established central role in mechanosensing and in mechanotransduction,^17^ transmitting—and translating—forces between the cytoskeleton and the ECM through mechanoresponsive adaptors (talin),^18,19^ and recruiting downstream signaling proteins (focal adhesion kinase, Src kinases).^20^ Increasing matrix stiffness enhances integrin clustering and focal adhesion formation, promoting downstream signaling through pathways such as RhoA and FAK that regulate cytoskeletal organization and cell proliferation.^21,22^ Mechanical cues from the ECM thus serve not only as structural constraints but also as active regulators of cell behavior through integrin mechanotransduction.

Here, we investigate the effect of extracellular acidosis on the regulation of microtubule dynamics via integrins. First, we assess the role of integrins in sensing acidosis and identify β1 integrin as a pH-sensitive receptor. We demonstrate that low extracellular pH increases β1 integrin engagement, resulting in decreased microtubule stability and polymerization duration by using molecular dynamics simulations, total internal reflection fluorescence (TIRF) microscopy, +TIP comet tracking, and a newly developed magneto-mechanical actuation device that allowed us to mimic different mechanical properties inherent to tissues.^23^ We then explore the mechanisms involved in this process, revealing that RhoA and its downstream effector Collapsing Response Mediator Protein-2 (CRMP-2) mediate the pH-dependent disruption of microtubule dynamics, which translates into impaired intracellular transport. This work points toward a new role for the β1 integrin/RhoA/CRMP-2 axis in the regulation of cytoskeletal dynamics by pH, providing a bridge between mechanosignaling and acidosis.

## 2 Results

### 2.1 Extracellular pH Disrupts Microtubule Dynamics via β1 Integrin

The microtubule cytoskeleton plays a core function in cells: it directs vesicle trafficking, establishes cell polarization, participates in focal adhesion turnover, and cellular mechanosensing and migration. This central role prompted us to investigate the effect of the extracellular pH on microtubule dynamics. To this end, Suit2 pancreatic ductal adenocarcinoma (PDAC) cells were transfected with fluorescently labeled end-binding protein 1 (EB1-RFP). EB1 is a +TIP protein that localizes to the (+) end of microtubules. Here, we used TIRF time-lapse microscopy to monitor the movement of EB1-rich comets to assess the dynamics of MT polymerization and growth (Figure 1A).

**Figure 1:**
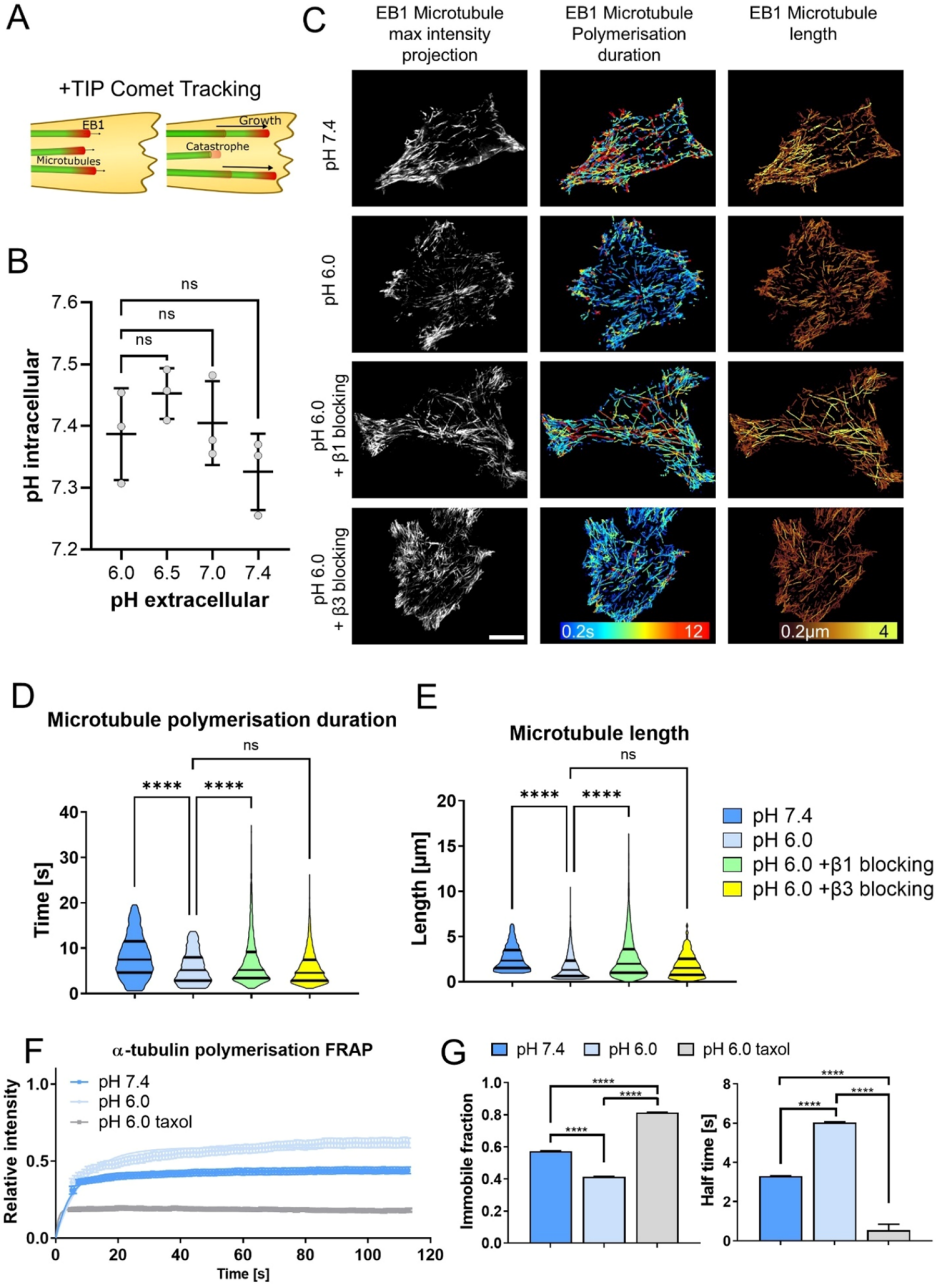
Extracellular acidosis reduces microtubule stability in an integrin-dependent manner. (A) Schematic of the EB1 comet tracking method. Fluorescently labeled +TIP proteins assemble at the (+) end of microtubule, tracking their growth, which can be readily extracted from particle tracking analysis. (B) Intracellular pH measured by fluorometric intracellular pH assay in Suit2 cells cultured at different extracellular pH. Mean ± SD, *n* = 3. No significant differences observed by one-way ANOVA with Dunnett’s multiple comparison post hoc test. (C) Maximum intensity projection for TIRF microscopy time-lapse tracking of microtubule (+) end polymerization (EB1-RFP comets), tracks of microtubules representing polymerization duration (jet scale), and length (solar scale). Suit2 cells were cultured in pH 7.4 and 6.0 with and without β1 integrin or β3 integrin blocking. β1 means β1 integrin, β3 means β3 integrin. Scale bar 10 μm. (D, E) Quantification of microtubule polymerization duration (track time, D) and length (track length, E) based on particle tracking analysis of (C). Violin plot represents the frequency distribution, median, and quartiles, *n* = 4461, 3864, 4761, and 6971 for pH 7.4, pH 6.0, pH 6.0 + β1 integrin blocking, and pH 6.0 + β3 integrin blocking, respectively. Markers (*) denote significance between indicated groups by one-way ANOVA followed by Dunnett’s multiple comparison post hoc test. ns—not significant, ****p < 0.0001. (F) Fluorescence recovery curves for α-tubulin-mCherry by FRAP in Suit2 cells cultured at pH 7.4, pH 6.0, or pH 6.0 + taxol. Mean fluorescence intensity (MFI) relative to prebleached intensity. (G) Quantification of the immobile fraction and the half-time based on the recovery curves on (F). Histogram bars represent mean ± SEM, *n* = 81, 43, and 68 for pH 7.4, pH 6.0, and pH 6.0 + taxol, respectively. Markers (*) denote significance between indicated groups by one-way ANOVA followed by Tukey’s post hoc test. ns—not significant, ****p < 0.0001.

Previous work had focused on the direct effect of the pH on microtubule dynamics *in vitro*. To verify that the effect observed is mediated by extracellular pH and is not driven by associated changes in intracellular pH, we used a fluorometric pH assay to quantify changes in pHi. No significant changes in pHi were observed in Suit2 (Figure 1B) or pancreatic stellate cells (Figure S1) in response to variations in extracellular pH (pHe) within the range explored here (pH 6.0–7.4).

Particle tracking analysis of the +TIP comets revealed a decrease in both microtubule polymerization duration and microtubule length at pH 6.0 compared to pH 7.4 (Figure 1C–E; Table S1), which is indicative of a decrease in MT stability. Similarly, fluorescence recovery after photobleaching (FRAP) of α-tubulin revealed a significant decrease in the immobile fraction and an increase in recovery half-time (*t*_1/2_) for cells cultured under acidosis (pH 6.0) compared to physiological pH (7.4), reflecting decreased microtubule stability and reduced MT polymerization dynamics (Figure 1F–G). To validate these findings, we used a low, sublethal dose of Taxol (10 nM) as a positive control for microtubule stabilization.^24^ At this concentration, Taxol acts as a kinetic stabilizer that suppresses microtubule disassembly without inducing the hyperpolymerization or drastic tubulin pool depletion associated with micromolar doses.

Taxol treatment increased the immobile fraction and significantly reduced half-time compared to both untreated conditions (Figure 1G). By kinetically ’locking’ the stable microtubule network and suppressing slow polymerization-driven turnover, the fluorescence recovery of the remaining mobile fraction is dominated by the fast diffusion of soluble tubulin dimers. This uncoupling of diffusion and microtubule dynamics confirms that the polymerization kinetics observed under acidosis result from impaired polymerization machinery.

Both β1 and β3 integrins have a pivotal role in the communication of cells with their environment.^25^ To elucidate their distinct roles in the mechanism of pH-dependent microtubule regulation, we used antibodies to functionally block their substrate (fibronectin) engagement (Figure 1C–E; Table S1). β1 integrin blocking abrogated the effect of low pH on microtubule dynamics, resulting in MT polymerization duration and length similar to pH 7.4 control, whereas β3 integrin blocking had no effect on MT regulation. These results indicate that β1 integrin and not β3 integrin is responsible for the modulation of MT stability in response to acidosis.

To further confirm the role of β1 integrin in the pH-driven modulation of microtubule dynamics, we assayed β1 integrin overexpressing (β1^+/+^) or integrin knockout (β1^–/–^) mouse epithelial (GE11) cells^25^ (Figure 2; Table S2). Consistent with the previous results obtained with Suit2 cells, β1^+/+^ GE11 cells showed a decrease in microtubule polymerization duration and length when cultured under acidic (pH 6.0) conditions in comparison to physiological (pH 7.4) conditions. However, this difference is not observed in β1^–/–^ GE11 cells, confirming the vital role of β1 integrin in the acidosis-dependent microtubule instability.

**Figure 2:**
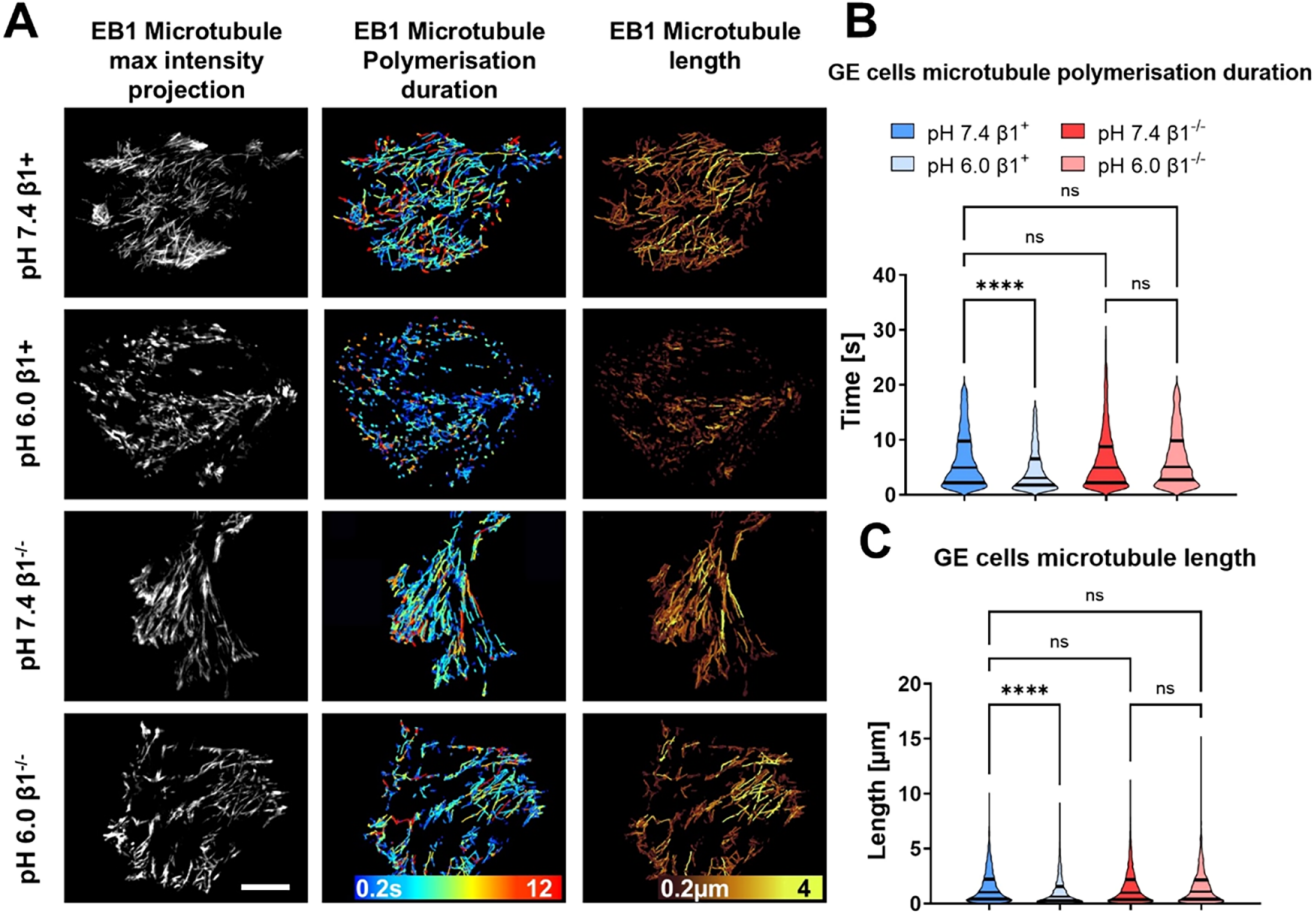
Acidosis-dependent microtubule instability requires β1 integrin expression. (A) Maximum intensity projection images generated from TIRF time‑lapse microscopy for tracking of microtubule (+) end polymerization (EB1-RFP comets), tracks of microtubules representing polymerization duration (jet scale), and length (solar scale). β1^–/–^ integrin (β1 integrin knockout) or β1 integrin expressing (β1+) mouse epithelial GE11 cells were cultured in pH 7.4 and 6.0. Scale bar 10 μm. (B) Quantification of microtubule polymerization duration (track time, B) and length (track length, C) based on particle tracking analysis of (A) Violin plot represents the frequency distribution, median and quartiles, *n* = 2856, 2561, 3689, and 2206 for pH 7.4 GE11 β1+, pH 6.0 GE11 β1+, pH 7.4 GE11 β1^–/–^, and pH 6.0 GE11 β1^–/–^, respectively. Markers (*) denote significance between indicated groups by one-way ANOVA followed by Dunnett’s multiple comparison post hoc test. ns—not significant, **** *p* < 0.0001.

### 2.2 Extracellular Acidosis and Mechanical Stimulation Synergistically Increase the Activation of β1 Integrin

Integrins are transmembrane receptors that convert extracellular signals into intracellular responses and are sensitive to both biochemical changes and physical cues in the microenvironment. β1 integrin plays a central role in mediating mechanotransduction in epithelial and carcinoma cells, and its activity is known to be regulated by ligand binding, divalent cation coordination, and cytoskeletal tension. Acidosis is a common characteristic of the extracellular matrix (ECM) across a range of tissues and physiological or pathological processes, including muscle activity,^26^ sepsis,^27^ bone resorption,^28^ cartilage homeostasis,^29^ and the tumor microenvironment.^30^ However, it is still unclear how acidic pH might interact with mechanical factors such as substrate stiffness or dynamic stretch to influence β1 integrin activation.

To examine this, we cultured Suit2 pancreatic cancer cells on fibronectin-coated PDMS substrates of defined stiffness (soft ∼ 1 kPa and stiff ∼ 10 kPa) under either physiological pH (7.4) or acidic pH (6.0). We also tested the effects of dynamic mechanical stimulation by applying uniaxial stretch (30 ± 5% strain for 15 min) to cells grown on soft PDMS using a magneto-mechanical actuation device (Figure 3A).^23^ Additional controls included treatment with 1 mM MnCl_2_ (30 min) at pH 7.4, known to promote integrin activation, and 2 mM EDTA (30 min) at pH 6.0 to inhibit activation by chelating divalent cations. A schematic overview of all conditions is shown in Figure 3B.

**Figure 3:**
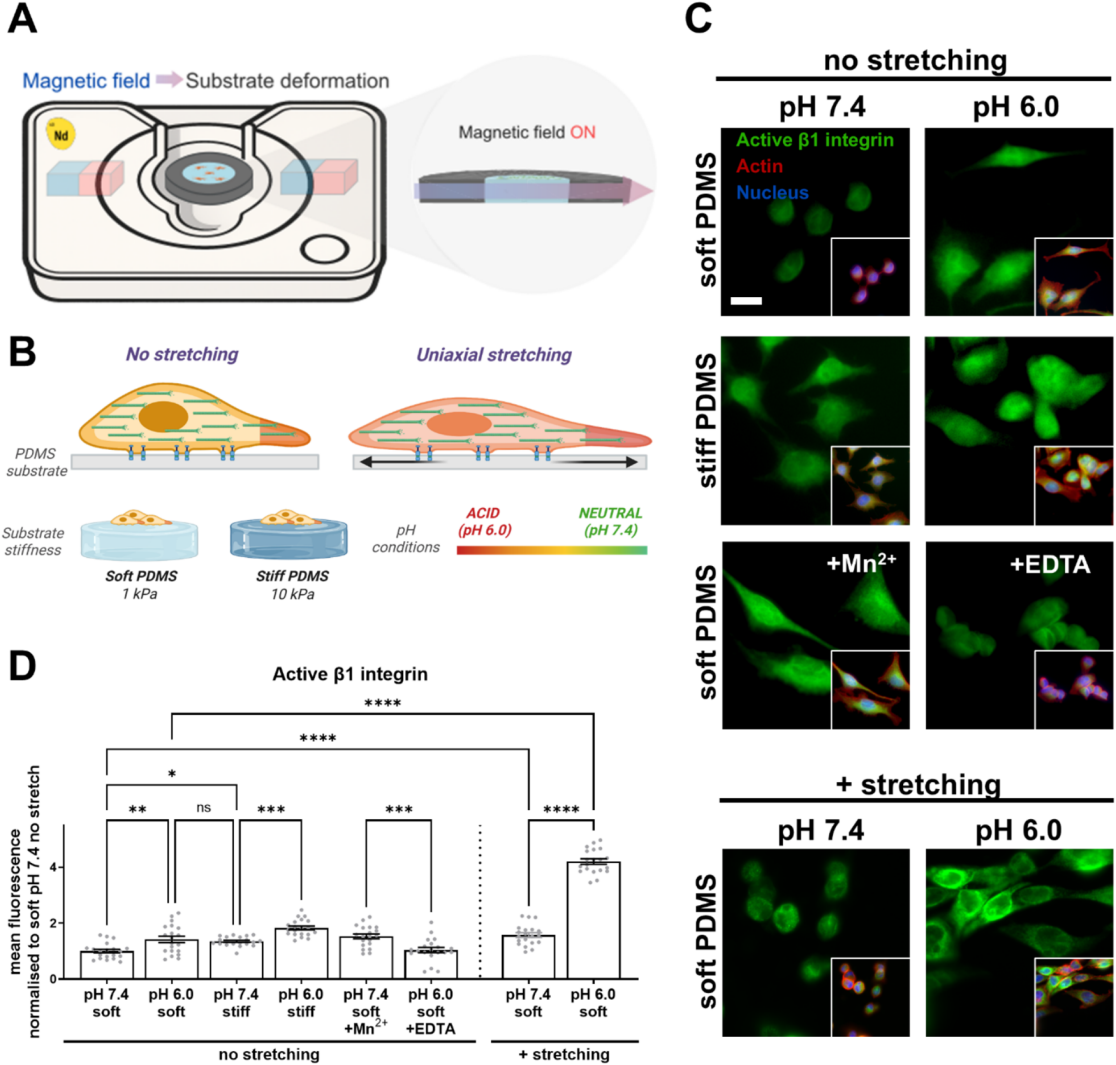
Extracellular acidosis and mechanical stimulation act in synergy to increase the activation of β1 integrin. (A) Diagram representing the magneto-mechanical actuation device used to achieve the uniaxial strain of the PDMS substrates. (B) Schematic illustration of the experimental conditions. (C) Wide-field microscopy images of active β1 integrin in Suit2 cells: cultured on unstretched soft (∼1 kPa) or stiff (∼10 kPa) fibronectin-coated PDMS elastomeric substrates, in pH 7.4 or pH 6.0. Cells were treated with 1 mM MnCl_2_ for 30 min (+Mn^2+^) and with 2 mM EDTA for 30 min (+EDTA) when indicated. Cells were stretched using uniaxial strains of 30 ± 5% in magnitude for 15 min (+stretching) when indicated. Scale bar 20 μm. (D) Quantification of mean fluorescence intensity of active β1 integrin signal presented in (C). Histogram bars represent mean ± SEM, dots represent individual data points; *n* = 20 cells. Markers (*) denote significance between indicated groups by one-way ANOVA followed by Kruskal–Wallis multiple comparison post hoc test. ns—not significant, * 0.01 < *p* < 0.05, ** 0.001 < *p* < 0.01, *** 0.0001 < *p* < 0.001, **** *p* < 0.0001.

To test the activation of β1 integrin, a monoclonal antibody that specifically recognizes the active β1 integrinconformation was used via immunofluorescence staining. Cells cultured in soft PDMS at pH 7.4 showed the lowest levels of β1 integrin activation (Figure 3C,D). Decreasing the pH to 6.0 increased β1 integrin activation (∼1.3-fold), and this effect of acidic pH was more pronounced in stiff PDMS (almost 2-fold). Uniaxial stretching induced β1 integrin activation in all conditions, with a notable increase of 4-fold for pH 6.0 and soft PDMS. Treatment with Mn^2+^ (β1 integrin activator) at pH 7.4 and soft PDMS induced activation of β1 integrins comparable to the activation seen by the effect of stretching. EDTA at pH 6.0 and soft PDMS abolished the acid-induced increase in β1 activation, consistent with the requirement of divalent cations for integrin activation.

### 2.3 Extracellular Acidosis Increases β1 Integrin Affinity for RGD-Containing Ligands in the Closed State by Displacing Ca^2+^ with Mg^2+^ at the ADMIDAS via Protonation of Asp138

To understand how low pHe would influence the structure–function of α5β1 integrin, we performed molecular dynamics (MD) simulations in microsecond time scale. First, we predicted the protonation state of the closed α5β1 headpiece at both physiological and acidic pH. The p*K*_a_ values for residues in α5β1 that have changed in protonation state are shown in Table S3.

The integrin headpiece is responsible for the recognition of extracellular ligands such as RGD-containing proteins. RGD binding is mediated by metal ions tightly coordinated by specific residues within the head, and it has been observed that the activation state of integrin can be modulated by exposing integrins to high concentrations of metal ions.^31^ To study the influence of pH on α5β1 integrin structure and function, we conducted comparative MD simulations using the corresponding protonation states with two different sets of metal ions bound to the three metal-ion-binding sites in the RGD-binding area. In our simulations, metal-ion-dependent adhesion site (MIDAS) was always occupied by Mg^2+^, while adjacent to MIDAS (AMIDAS) and the synergistic metal binding site (SyMBS) were both occupied by either Ca^2+^ or Mg^2+^. ADMIDAS regulates ligand-binding affinity and is coordinated by Asp137 and Asp138.^31,32^ In pH 6.0, Asp138 is predicted to be protonated (Table S3) and, as a result, becomes noncharged. Our simulations revealed that Ca^2+^ dissociates from the ADMIDAS site within 1 μs upon protonation of Asp138 in all three MD replicas that we conducted. However, when Mg^2+^ was bound at the ADMIDAS site, it did not dissociate after protonation of Asp137, presumably because its tight interaction with Asp137 was enabled by Mg^2+^’s ability to strongly bind carboxyl oxygens (Figure 4 and Figure S2). In our simulations, protonated Asp138 did not coordinate ADMIDAS ion well (Table S3) because of the neutral charge of its side chain. This led to changes in coordination of the metal ions, where Asp259 moved from MIDAS closer to ADMIDAS Mg^2+^ ion, increasing the positive potential in the environment of MIDAS and promoting greater electrophilicity of MIDAS Mg^2+^ for RGD-containing ligands in the closed state. In summary, our results suggest that at pH 6.0, ligand-binding affinity of β1 integrin increases due to the replacement of Ca^2+^ by Mg^2+^ at ADMIDAS, controlling the partial charge of the MIDAS ion.

**Figure 4:**
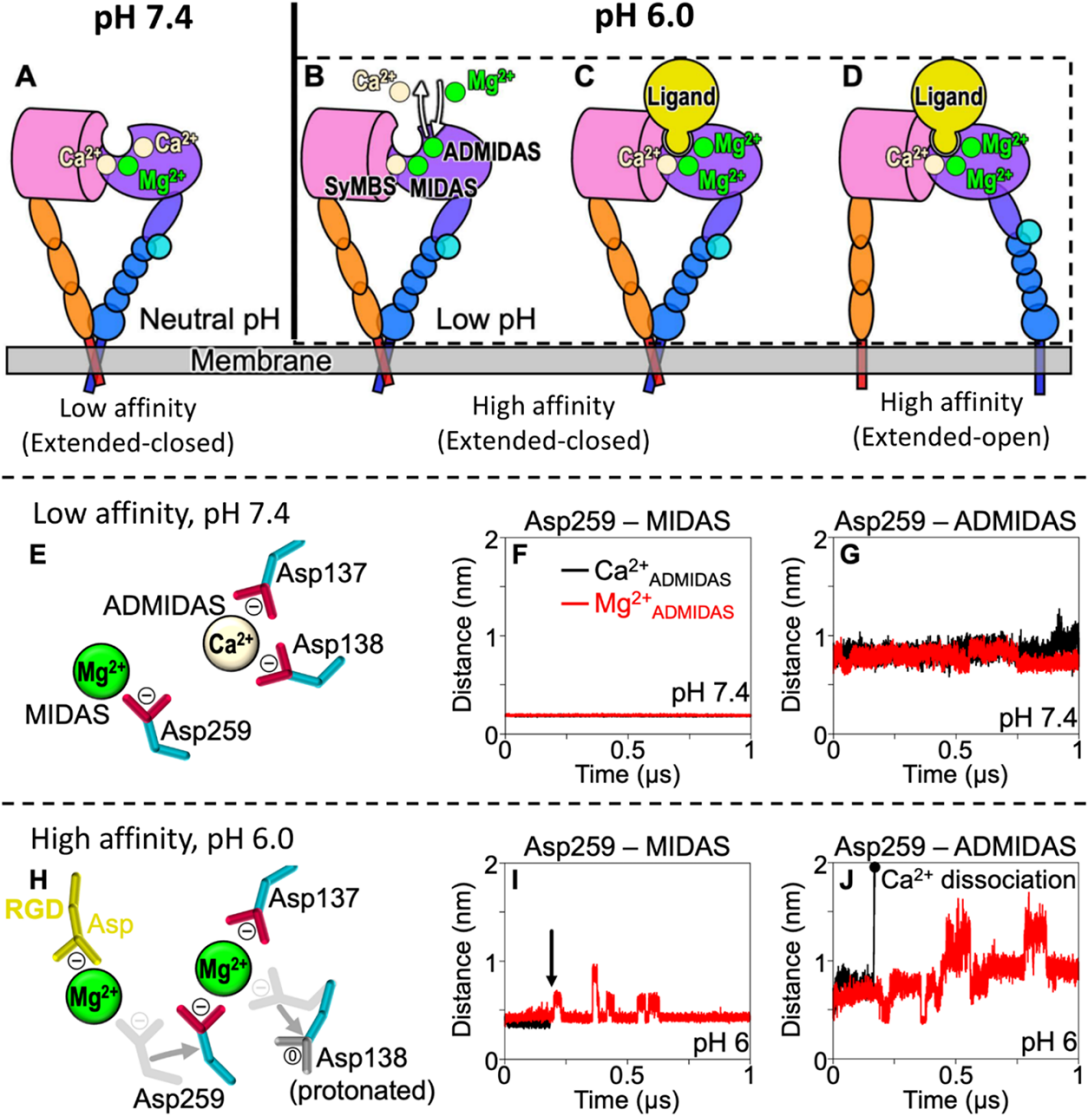
Low pH activates α5β1 integrin by increasing affinity for RGD-containing ligands in the closed state by favoring displacement of Ca^2+^ with Mg^2+^ at the ADMIDAS via protonation of Asp138. Schematic representation of (A) low-affinity extended-closed conformation at pH 7.4, and (B) replacement of Ca^2+^ with Mg^2+^ at the ADMIDAS under pH 6.0 in extended-closed state leading to high affinity, (C) recruitment of ligand, (D) stabilization of the liganded integrin in extended-open conformation. Schematic representation of coordination of the MIDAS and ADMIDAS by Asp259, Asp137, and Asp138 (E) under pH 7.4, forming a low-affinity state, and (H) pH 6.0, forming a high-affinity state. Distance from the closest oxygen atom of Asp259 to (F, I) MIDAS and (G, J) ADMIDAS metal ion in low-affinity pH 7.4 and high-affinity pH 6.0 conditions, respectively. Under pH 6.0, Asp138 becomes protonated and Ca^2+^ ion dissociates from the ADMIDAS site during the 1 μs MD. (I, J) After Ca^2+^ dissociation, the MD simulation was terminated (shown with the arrow in I).

Mn^2+^ is often used to activate integrins, and recent studies have demonstrated that α5β1 integrin activation requires bound ligand for stabilization of the extended-open state. Intact α5β1 is predominantly in the bent-closed state in Mn^2+^ in the absence of ligand, and its opening is induced by ligand-binding.^33^ This suggests that the closed state of integrin becomes high-affinity for ligand before the headpiece opening, in the closed state, where affinity is regulated by ADMIDAS, which is responsible for stabilizing integrin in discrete states.^31^ Higher concentrations of Ca^2+^ metal ion inhibit integrin,^32^ while double α5β1 D137A and D138A mutant cannot be inhibited with Ca^2+^.^31^ This suggests that Ca^2+^ inhibits integrin via ADMIDAS, while the binding of Mg^2+^ at the ADMIDAS increases affinity for RGD-containing ligands. The explanation is that, similarly to the Mn^2+^ ion, the Mg^2+^ ion strongly favors carboxyl over carbonyl oxygens, while Ca^2+^ coordinates well with both.^31^ Thus, both Mn^2+^ and Mg^2+^ increase affinity for ligands by favoring the movement of Asp259 side chain from MIDAS to ADMIDAS, making MIDAS more positive and ready to bind ligand with high affinity via Asp in RGD motif. Our MD results demonstrate that acidic pH (6.0) activates α5β1 integrin via protonation of Asp138 in the closed state, which favors the displacement of Ca^2+^ by Mg^2+^ at the ADMIDAS and enhances interaction between Asp259 and ADMIDAS Mg^2+^.

Taken together, these results suggest that low extracellular pH disrupts microtubule dynamics through the allosteric modulation of β1 integrin activation. Immunofluorescence analysis of active β1 integrin with TIRF revealed an increase in the average focal adhesion (FA) area at pH 6.0 versus pH 7.4 (Figure S3A) with dimensions comparable to cells treated with Mn^2+^, a known integrin activator (Figure S3D). Treatment with EDTA, a chelating agent that sequesters the divalent cations required for integrin activation, inhibited the effect of acidosis on FA area, resulting in values similar to pH 7.4 control (Figure S3D). In accordance with MD simulations, these results indicate higher β1 integrin activation in response to acidosis.

### 2.4 Extracellular Acidosis Increases RhoA Activation

RhoA is a small Rho GTPase that governs cellular mechanotransduction pathways by coordinating the structure, the function, and the dynamics of the cytoskeleton. RhoA is found downstream of a variety of signaling cascades, including G-protein-coupled receptors—GPCRs (Gs, Gq)^34^ and integrins, rapidly switching between inactive (RhoA-GDP) and active (RhoA-GTP) states.^35^ Notably, RhoA is activated in response to integrin-mediated mechanosensing of substrate stiffness, among other mechanical stimuli, which, in turn, regulates the polymerization and contractility of actomyosin.^36^ This central role in mechanosignaling and cytoskeletal regulation, together with our previous findings on the role of β1 integrin in extracellular pH sensing, positions RhoA as an intermediary candidate in the regulation of microtubule dynamics by acidosis.

To analyze the effect of extracellular pH on RhoA activation, we used a molecular fluorescence resonance energy transfer (FRET) sensor. FRET is a fluorescence microscopy technique widely used to study protein–protein interactions: it relies on the transfer of energy between a donor and an acceptor fluorophore pair, which is heavily dependent on the distance between them. The RhoA FRET sensor used here is designed to undergo a conformational change upon RhoA activation (GTP loading) that brings the donor/acceptor pair into close proximity, enabling FRET between them (Figure 5A). Analysis of donor emission intensity after acceptor photobleaching (Figure 5B,C) revealed a significantly higher FRET efficiency in Suit2 cells cultured at pH 6.0 compared to pH 7.4, which is indicative of higher RhoA activity. The increase in RhoA activation was accompanied by an increase in traction force generation (Figure S4) measured by elastic micropillar arrays - an effect observed downstream of RhoA activation.^15^

**Figure 5:**
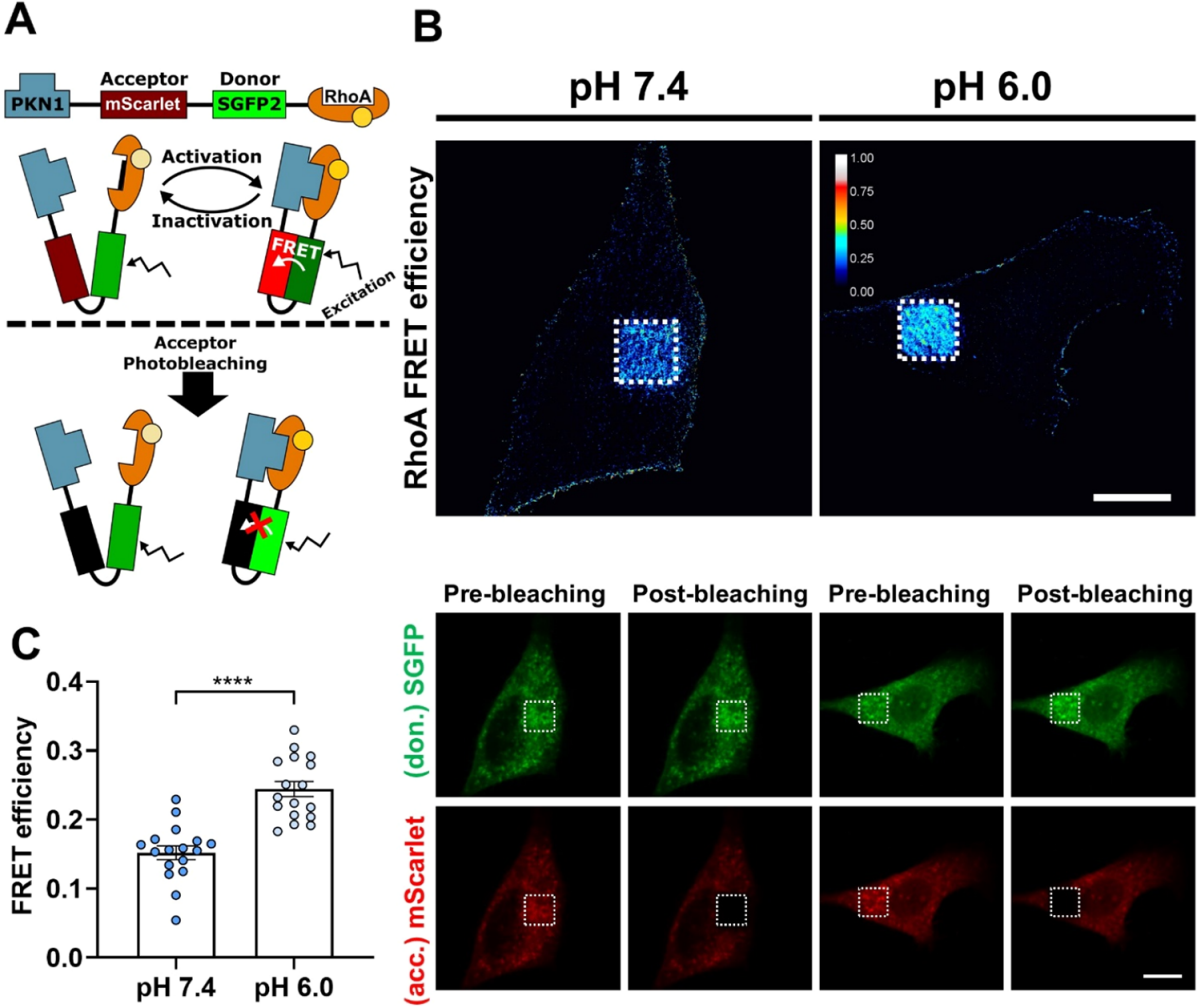
Extracellular acidosis activates RhoA. (A) Schematic of the RhoA (mScarlet-SGFP2) FRET sensor used to quantify RhoA activity. In the inactive (RhoA-GDP) or open configuration, the large distance between the donor and the acceptor prevents efficient energy transfer via FRET. Upon RhoA activation (RhoA-GTP), the PKN1 domain binds to RhoA-GTP, the FRET pair is brought into close proximity and energy transfer occurs from the exited donor to the acceptor. In this configuration, the energy transfer diminishes the fluorescence emission intensity from the donor. Photobleaching of the acceptor blocks FRET, increasing the donor’s fluorescence emission intensity. In the open (inactive) configuration (low FRET state), photobleaching of the acceptor has no effect on the donor’s emission intensity. (B) Ratio of donor emission intensity before and after acceptor photobleaching in Suit2 cells cultured at pH 7.4 and pH 6.0. (C) Quantification of the acceptor photobleaching FRET efficiency—ratio obtained by subtracting the donor-emitted signal intensity before the photobleaching of the acceptor from its intensity after photobleaching the acceptor, normalized to the donor signal intensity post-photobleaching. Histogram bars represent mean ± SEM, dots represent individual data points; *n* = 17 cells. Markers (*) denote significance by two-tailed unpaired *t* test, **** *p* < 0.0001.

### 2.5 RhoA Activation Modulates Microtubule Dynamics via ROCK/CRMP-2 Pathway

Our results indicate that the pH-dependent activation of β1 integrin translates to an increase in RhoA activation, linking acid-sensing to microtubule dynamics. RhoA can regulate microtubule stability through its downstream effectors ROCK1 and ROCK2, both members of the Rho kinase (ROCK) family. ROCK family has several targets, including the Collapsin Response Mediator Protein-2 (CRMP-2), a member of the dihydropyrimidinase-like or Collapsin Response Mediator Protein (CRMP) family that plays key roles in microtubules dynamics. CRMP-2 binds to the α/β tubulin heterodimer, promoting MT polymerization and assembly, while ROCK-dependent phosphorylation/inactivation of CRMP-2 at Thr555 mediates microtubule collapse^37–39^ (Figure 6A,B).

**Figure 6:**
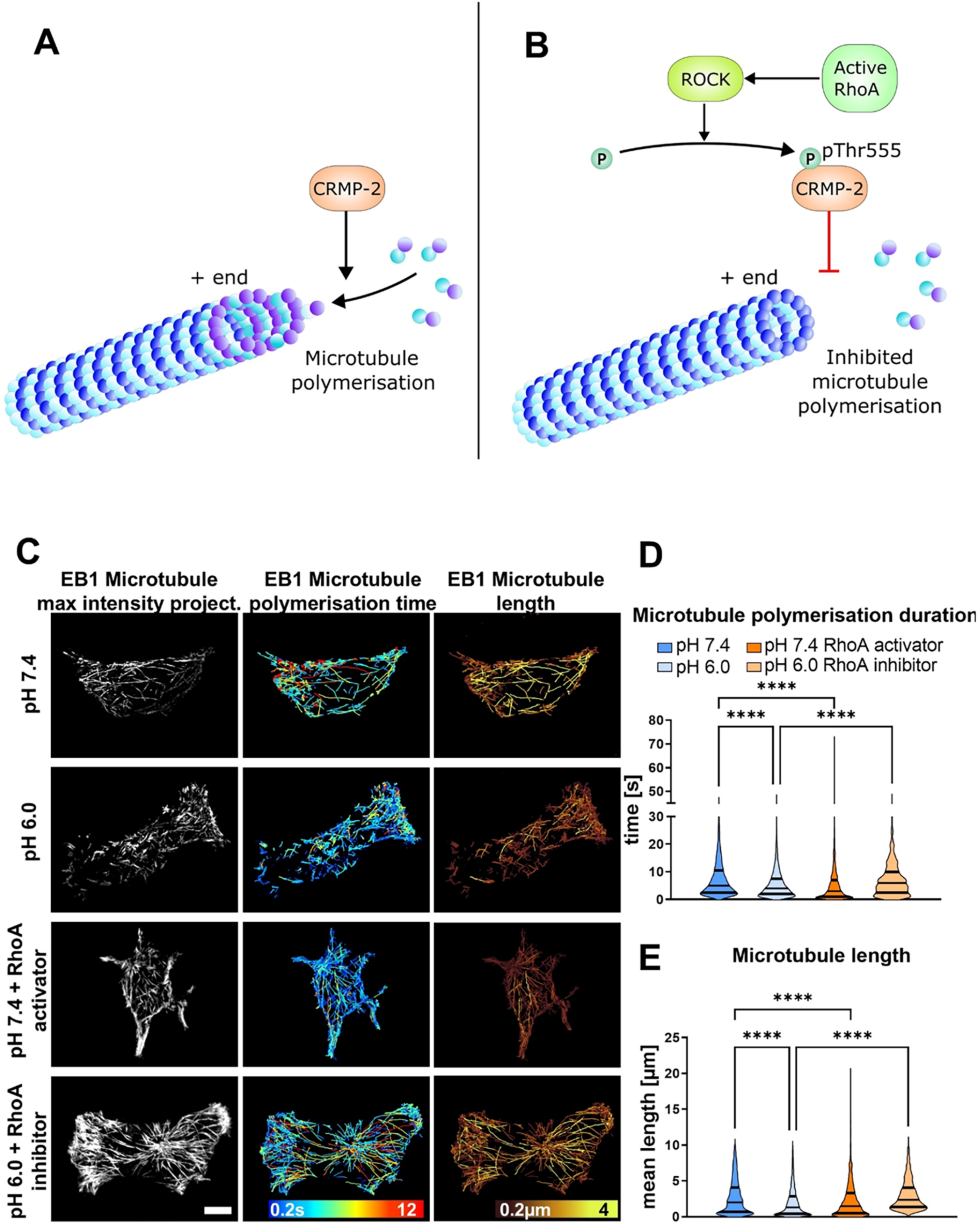
Extracellular acidosis regulates microtubule stability via RhoA. (A) Model that illustrates the role of CRMP-2 in microtubule polymerization, and (B) RhoA/ROCK-mediated phosphorylation of CRMP-2 at position Thr555 that inactivates CRMP-2 and induces microtubule collapse. (C) Maximum intensity projection images generated from TIRF time‑lapse microscopy for tracking microtubule (+) end polymerization (EB1-RFP comets), tracks of microtubules representing polymerization duration (jet scale), and length (solar scale). Suit2 cells are either cultured in serum free media or treated with RhoA inhibitor (CT04) or activator (CN01). Scale bar 5 μm. (D, E) Quantification of the microtubule duration (track time, B) and length (track length, C) based on particle tracking analysis of (A). Violin plot represents the frequency distribution, median, and quartiles, *n* = 1811, 1972, 2678, and 1787 for pH 7.4, pH 6.0, RhoA inhibitor (CT04), or activator (CN01), respectively. Markers (*) denote significance from pH 6.0 by the Kruskal–Wallis test followed by Dunn’s multiple comparison test. **** *p* < 0.0001

We studied microtubule dynamics of Suit2 cells at pH 7.4 and 6.0 with or without RhoA activator or inhibitor. Activating the RhoA pathway in cells at pH 7.4 induced similar levels of microtubule polymerization as those displayed in cells at pH 6.0. Inhibition of RhoA in cells cultured at pH 6.0 restored microtubule dynamics observed at pH 7.4 (Figure 6C–E).

To elucidate the potential regulatory mechanism, we used immunoblotting to investigate the levels of total and phosphorylated CRMP-2 (pCRMP-2) in Suit2 cells cultured at pH 7.4 and 6.0 (Figure 7A–C and Figure S5). According to the manufacturer, CRMP-2 and pCRMP-2 show up at around 62 and 70 kDa band sizes, respectively. The total levels of CRMP-2 were found to be constant at both pH values and in the presence of a potent RhoA activator. Without the presence of the RhoA activator, the levels of pCRMP-2 were not detectable, but when the RhoA activator was present, the pCRMP-2 levels were significantly higher at pH 6.0 than those at pH 7.4.

**Figure 7:**
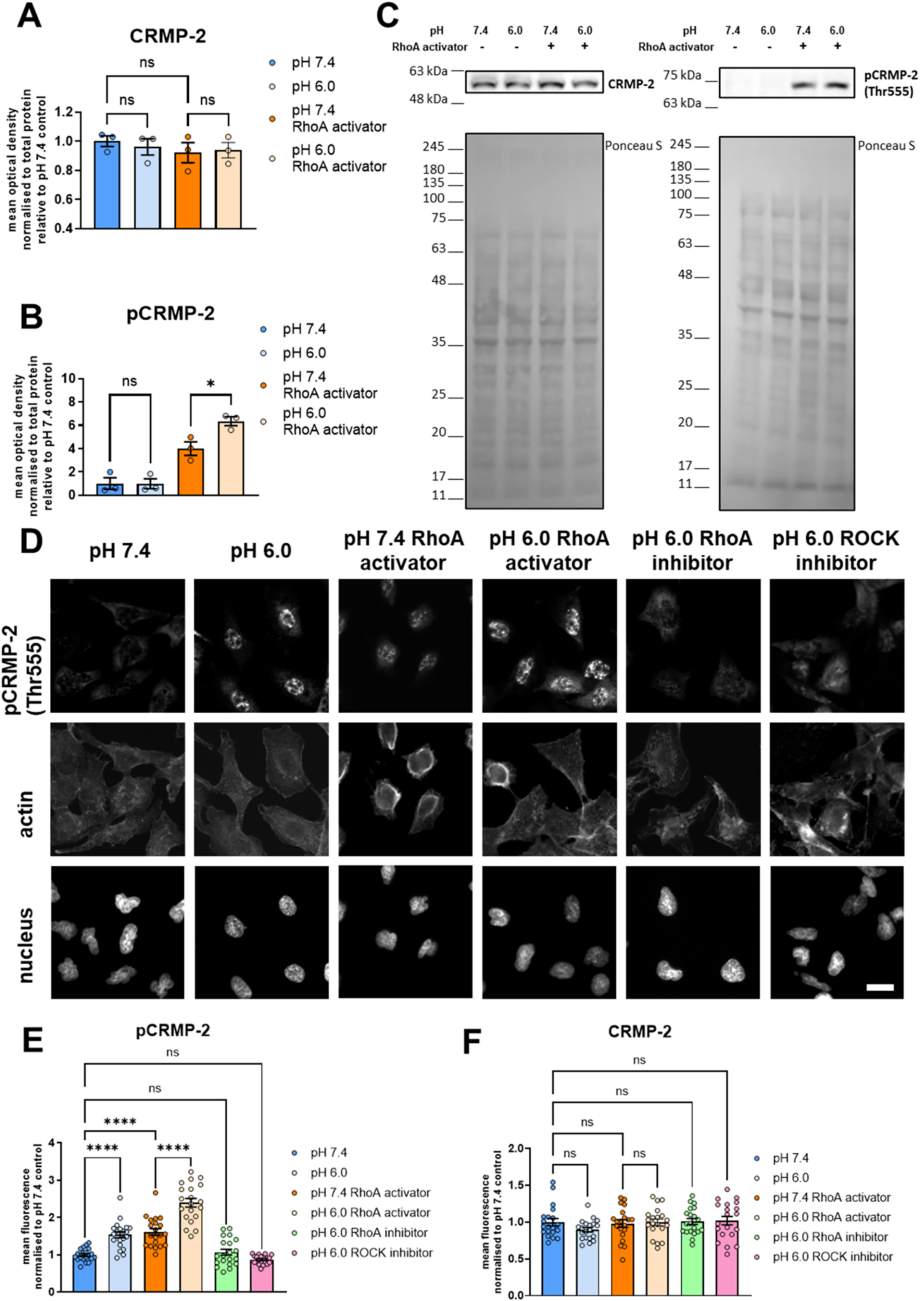
CRMP-2 is phosphorylated in acidosis in a RhoA-dependent manner. Protein expression quantified using Western blot optical density for (A) total and (B) phosphorylated on Thr555 CRMP-2, normalized to total protein Ponceau S staining, relative to pH 7.4. *n* = 3 experimental replicates for pH 7.4, pH 6.0, pH 7.4 with RhoA activator (CN01), and pH 6.0 with RhoA activator (CN01). Histogram bars represent mean ± SEM (C) Representative cropped images of blots shown in full in Figure S5. (D) Wide-field microscopy images for pCRMP-2 (Thr555) in immunostained Suit2 cells at pH 7.4, pH 6.0, pH 7.4 with RhoA activator (CN01), pH 6.0 with RhoA activator (CN01), pH 6.0 with RhoA inhibitor (CT04), and pH 6.0 with ROCK inhibitor (Y-27632). Scale bar 10 μm. (E) Quantification of mean fluorescence intensity of pCRMP-2 (Thr555) signal presented in (D). (F) Quantification of mean fluorescence intensity of CRMP-2 signal presented in Figure S6. Histogram bars represent mean ± SEM, dots represent individual data points; *n* = 20 cells. Markers (*) denote significance between indicated groups by one-way ANOVA followed by Kruskal–Wallis multiple comparison post hoc test. ns—not significant, * 0.01 < *p* < 0.05, **** *p* < 0.0001.

To validate these results, we used immunofluorescence to visualize and quantify the levels of total and phosphorylated CRMP-2 in Suit2 cells cultured at pH values of 7.4 and 6.0. As expected, the total levels of CRMP-2 did not change with pH or with the presence of the RhoA inhibitor/activator or ROCK inhibitor (Figures S6 and 7F). We observed a marked increase of around 1.5-fold in the levels of pCRMP-2 in cells cultured at pH 6.0 compared to those at pH 7.4. The use of the RhoA activator at both pH values increased around 1.5-fold the levels of pCRMP-2 in comparison to the pCRMP-2 values for the same pH without the RhoA activator. Activating RhoA at pH 7.4 rescued the levels of pCRMP-2 observed at pH 6.0. Inhibiting RhoA or ROCK in cells at pH 6.0 decreased the levels of pCRMP-2 to the values seen in cells at pH 7.4 (Figure 7D–E).

It has been previously reported that the increased expression of pCRMP-2 and its nuclear localization are associated with breast cancer progression.^40^ Interestingly, Suit2 cells cultured at pH 6.0 expressed a larger amount of pCRMP-2, and it is predominantly localized in the nucleus (Figure 7D and Figure S7).

### 2.6 Extracellular Acidosis Regulates Golgi Complex Localization and Architecture

One of the central functions of the microtubule cytoskeleton is its role in the intracellular transport and organelle distribution. MTs serve as polarized tracks to direct the flow of traffic between the nucleus and the cell periphery. Intracellular vesicles, protein complexes, and organelles are transported along MTs by two unrelated families of MT-associated motor proteins: kinesins, which transport cargo toward the (+) end (anterograde), and dyneins, which transport cargo toward the (−) end (retrograde).

To investigate how the decrease in microtubule stability (polymerization duration and length) translates to their role in intracellular transport and organization, we analyzed the positioning of the Golgi apparatus within cells. The Golgi apparatus is a network of membranous cisternae and vesicles that participates in the folding, sorting, and trafficking of proteins between the endoplasmic reticulum (ER) and the plasma membrane. The Golgi complex is closely associated with the microtubule cytoskeleton and the centrosome, and its localization is governed by the interplay between (+) end- and (−) end-directed microtubule motor proteins.^41^ Disruptions in MT-mediated transport, therefore, correlate with changes in the distribution of the Golgi complex.

Suit2 cells were transfected with Golgi7 (β-1,4-galactosyltransferase)-GFP, a protein that localizes to the membrane of the Golgi complex and its associated vesicles (Figure 8A). We observed a marked decrease in the distance between the Golgi complex and the nucleus (centroid-to-centroid distance) (Figure 8B) as well as the spread of the Golgi complex (area), in response to acidic pH (6.0) compared to physiological pH (7.4) (Figure 8C), indicative of impaired microtubule-mediated transport. Conversely, β1 integrin blocking abrogated the effect of extracellular pH on Golgi localization, in agreement with our previous findings, which confirms the role of integrins in this pathway (Figure 8D).

**Figure 8:**
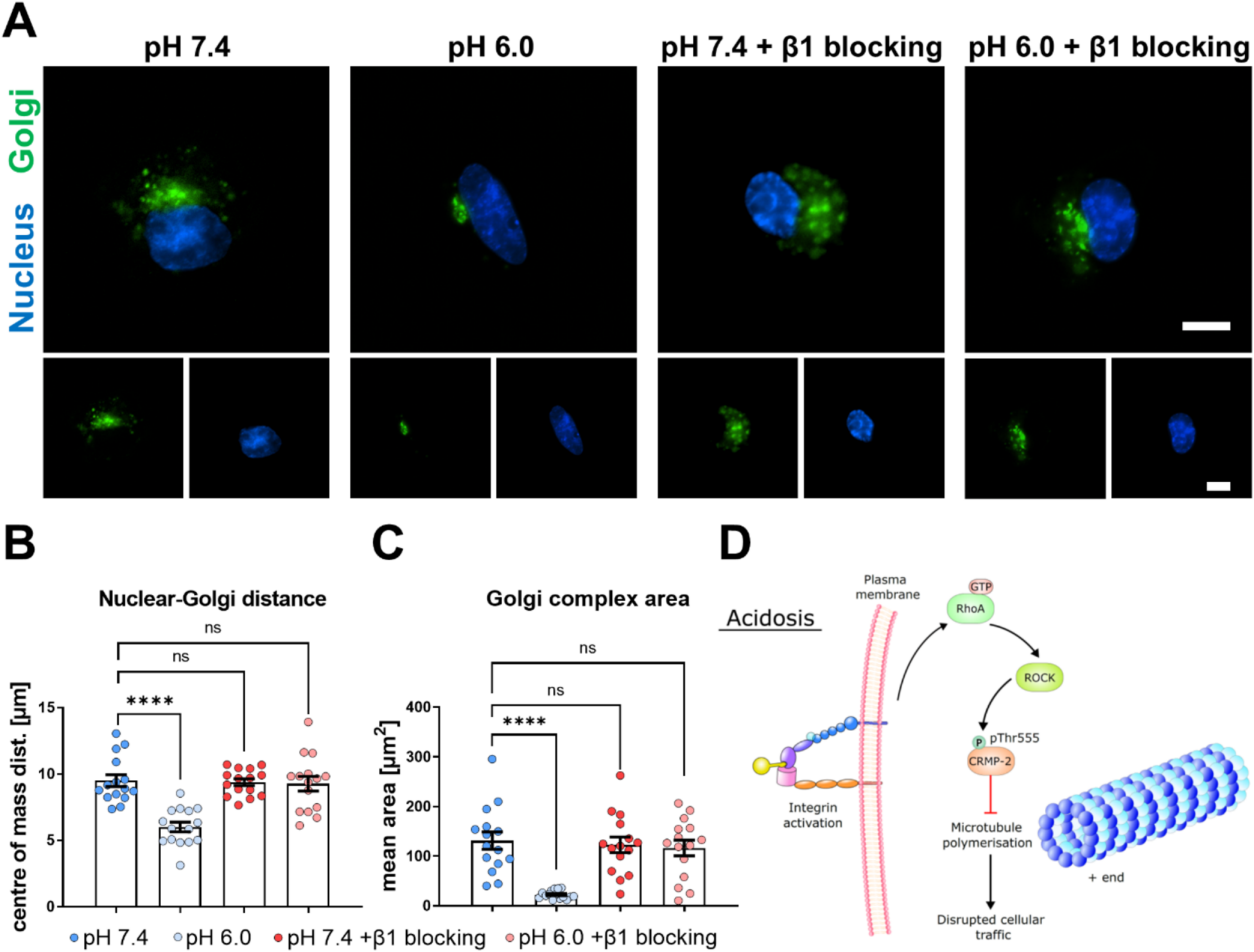
Extracellular acidosis regulates Golgi complex localization and architecture. (A) Epifluorescence images of the Golgi complex (identified by β-1,4-galactosyltransferase 1-GFP, green) and the nucleus (Hoechst 33342, blue) in Suit2 cells cultured at pH 7.4 and 6.0 with and without β1 integrin or β3 blocking. β1 means β1 integrin, β3 means β3 integrin. Scale bar 10 μm. (B) Quantification of the distance between the nucleus and the Golgi apparatus in (A), measured as the geometric distance between the centers of mass (intensity-weighted centroid) of the two structures. (C) Quantification of the area of the Golgi complex in (A). Histogram bars represent mean ± SEM, dots represent individual data points; *n* = 15 cells. Markers (*) denote significance from pH 7.4 by one-way ANOVA followed by Dunnett’s multiple comparison post hoc test. ns—not significant, ****p < 0.0001. (D) Model illustrating the molecular mechanism for the regulation of microtubule dynamics by acidosis.

## 3 Discussion

A variety of cell surface receptors have been associated with the response to acidosis, including proton-sensitive G protein-coupled receptors (GPCRs), acid-sensing ion channels (ASICs), and transient receptor potential (TRP) channels. Our results here indicate that the pH-dependent disruption of microtubule dynamics is mediated by β1 integrin, a mechanoreceptor implicated in cell-ECM adhesion and mechanosensing. We observed that cells integrate the biochemical (pH) and mechanical properties of the ECM (stiffness and stretching) to tune cytoskeletal dynamics via β1 integrin. Low pH, elevated stiffness, and stretching are hallmarks of many physiological processes such as inflammation and cancer development, and β1 integrin may represent a valuable target to harness them. This new role of integrins in pHe sensing has been proposed before but remained largely unexplored and not related to microtubule dynamics.^42^ Using MD simulations, we found that acidosis increases ligand-binding affinity of β1 integrin. Enhanced ligand binding likely accelerates β1 integrin activation. These findings support the hypothesis that β1 integrin acts as a pH sensor. In addition to integrins, several acid-sensing GPCRs (GPR4, GPR68, GPR65, and GPR123) may positively or negatively modulate RhoA concomitantly with β1 integrin,^34,43^ creating a network in which several receptors converge on a common cytoskeletal program.

Our results indicate that low pHe affects microtubules dynamics by impairing polymerization, which may compromise the formation and stability of the mitotic spindle. Mutations in microtubule-associated proteins (MAPs), such as the (+) end-binding protein APC, can increase the propensity for cancer,^44^ and aberrant upregulation of the MT destabilizing protein stathmin correlates with metastasis and EMT.^45,46^ We have previously reported that extracellular acidosis promotes cell proliferation in Suit2 (PDAC) cells,^47^ along with an increase in YAP and HIF-1A signaling. We hypothesize that this combination of an increased proliferation rate and spindle dysfunction could result in chromosomal instability, contributing to cancer progression. Investigating microtubule growth and stability dynamics in the mitotic spindle, however, has been restricted by the difficulty of detecting and monitoring individual microtubules in such a dense array.^48^ Recent advancements in 3D live cell imaging via lattice light-sheet microscopy (LLSM) have enabled researchers to resolve and analyze individual MTs within the mitotic spindle.^49^ Further research into the effect of acidosis on the stability and organization of the mitotic spindle may shed new light on the mechanisms by which metabolic dysregulation contributes to chromosomal instability and potentially to oncogenesis.

We found that the effect of acidosis on the microtubule cytoskeleton is mediated by the RhoA/ROCK/CRMP-2 axis, a central pathway in mechanosignaling and actomyosin regulation (Figure 8D). It is important to note that while immunofluorescence analysis successfully captured the acidosis-induced increase in CRMP-2 phosphorylation, these differences were not readily detectable by Western blotting under basal conditions (Figure 7B,C). This discrepancy arises from the inherent sensitivity limitations of Western blotting in detecting low-abundance and limited dynamic range, rather than indicating a lack of biological significance. The differences in CRMP-2 phosphorylation were observed following pharmacological RhoA activation, which increased pCRMP-2 to levels detectable by immunoblotting and revealed the pH-dependent regulatory effect. This pH-sensitive pCRMP-2 upregulation in the absence of the RhoA activator was further supported by complementary immunofluorescence imaging (Figure 7D,E). Moreover, we observed that the levels of expression and nuclear localization of pCRMP-2 significantly increased in low extracellular pH or acidosis, which phenocopies the behavior of pCRMP-2 in breast cancer.^40^ Our work positions the RhoA/ROCK/CRMP-2 axis as a key mediator in the interaction between acidosis and mechanosignaling and a potential therapeutic target in acidosis-driven metastasis.

Tumor acidosis (low extracellular pH) emerges from the metabolic shift toward anaerobic glycolytic (Warburg effect),^13^ which produces excess lactate, combined with the upregulation of lactate and H^+^ transporters and the conversion of excess CO_2_ into HCO3^–^ and H^+^ by carbonic anhydrases (CAIX).^50,51^ While acidosis has been regarded as a byproduct of tumor growth and aberrant metabolism, its role in cancer development is becoming increasingly relevant. Tumor acidosis can promote autophagy and immune evasion and drive metabolic reprogramming, migration, and invasion.^50,52^ We recently reported that acidosis, in combination with matrix rigidity, promotes the expression of master regulators in metabolism and survival (HIF1A), and mechanotransduction (YAP).^47^ Despite the emergent role of tumor acidosis in the regulation of cancer cell behavior, the underlying molecular mechanisms remain unexplored. Here, we found that extracellular pH modulates microtubule dynamics, which could have important consequences for intracellular organization, cell polarization, vesicular trafficking, nucleocytoplasmic shuttling, and cell division.

MTs represent a highly attractive therapeutic target in cancer due to their pivotal role in cell division. Indeed, numerous microtubule-targeting drugs (MTDs) are used in chemotherapy, including taxanes, colchicine, and vinca alkaloids (vinblastine), for their ability to interfere with cell cycle progression.^53^ However, MT dysregulation in cancer can also contribute to metastasis, migration, trafficking, and polarization. Understanding how tumor acidosis modulates MT dynamics may therefore shed new light on the crosstalk between cancer cells and the tumor microenvironment and open new opportunities to reposition MTDs to interfere with metastatic invasion.

An aspect of extracellular acidosis that remains controversial is its effect on intracellular pH (pHi). While it is widely accepted that cancer cells present a slightly alkaline pHi (>7.4) compared to normal epithelial cells (∼7.2),^54^ some studies have reported that a extracellular acidosis is accompanied by cytoplasmic acidification.^55,56^ Here we report no significant changes in pHi as a result of decreasing pHe, with pHi values in the expected range of 7.3–7.6. Indeed, the correlation between changes in extracellular and intracellular pH seems to be largely cell/tumor-type-dependent, likely due to differences in the expression of H^+^ extruders (NHE1, MCTs) and carbonic anhydrases (CAs).

While our data suggest a stable bulk intracellular pH, cancer cells are known to manipulate pH gradients at a subcellular level to drive key functions like invasion.^57^ This localized regulation is critical for the activity of invadopodia, which are membrane protrusions supported by an actin-rich core and associated microtubules that secrete proteolytic enzymes (e.g., matrix metalloproteinases or MMPs) to facilitate ECM degradation and cancer cell invasion.^58,59^ Tumor acidosis and pH are critical in the regulation and function of invadopodia: Both the sodium–proton exchanger (NHE1) and the carbonic anhydrase CAIX redistribute to invadopodia, contributing to local acidification of the extracellular space.^60,61^ In turn, this pericellular acidosis promotes the secretion and proteolytic activity of MMPs and cathepsins, cleaving ECM fibers to permit invasion. In light of our findings on the response of β1 integrin to acidosis, this pericellular decrease in pHe around invadopodia could also trigger local β1 integrin engagement, coordinating ECM remodeling and cell-ECM adhesion to direct cell migration. Simultaneously, the export of H^+^ by NHE1 results in the alkalinization of the pHi in the invadopodia. This alkalinization promotes actin polymerization by dissociating cofilin and cortactin.^62^ Further research will be required to elucidate the role of β1 integrin/RhoA in invadopodia, and to disentangle the effect of local vs global changes in both intra- and extracellular pH.

Perhaps, the more direct effect of tumor acidosis related to the disruption of MT dynamics may be the dysregulation of intracellular transport. In particular, extracellular acidosis has been found to regulate lysosome trafficking,^63,64^ which is mediated by microtubules. Microtubules are also involved in the transport and delivery of MMP-14 to invadopodia.^65^ We observed that extracellular acidosis decreases the distance between the nucleus and the Golgi apparatus, as well as the spread of the Golgi and its associated vesicles, which is consistent with altered MT transport. LAMP-2, an important protein in autophagosome maturation and lysosome stability, is upregulated by acidosis,^66^ which may be a key factor contributing to autophagocytosis and cancer cell survival. However, the implications of extracellular acidosis for intracellular transport, or potential implications on chemotherapeutic intake and metabolism, remain unexplored. Extending this analysis to stroma cells of PDAC could broaden the effects of extracellular acidosis on the tumor microenvironment: by the induction of myofibroblasts through the activation of β1 integrin, or the effect on ECM protein secretion through intracellular transport dysregulation.^67^ While more research will be required to understand how MT disruption affects intracellular regulation, our work highlights the role of the cytoskeleton and integrin-mediated cell-ECM adhesion in response to acidosis.

## 4 Materials and Methods

### 4.1 Experimental Design

The main objective of this study was to demonstrate the role of extracellular pH in changing the dynamics of microtubules and to elucidate the role of β1 integrin/RhoA/CRMP-2 signaling pathway mediating this effect. We studied microtubule polymerization and the involved pathway in physiological and acidosis conditions in a model Suit2–007 cancer cell line, without and with pharmacological agents (β1 integrin blocking antibodies, β3 integrin blocking antibodies, ROCK inhibitor Y027632, RhoA activator CN01, or RhoA inhibitor CT04), or in epithelialGE11 cells expressing β1 integrin or with β1 integrin knockout. Suit2–007 and pancreatic stellate cells (PSCs) were used to measure the intracellular pH values in different extracellular pH. Studies were also conducted *in silico* using molecular dynamics simulations for α5β1 integrin.

### 4.2 Cell Culture, Reagents, and Antibodies

Suit2–007, GE11, and PSCs cells were cultured in low glucose Dulbecco’s modified Eagle’s media (Cat. No. D6046, Sigma-Aldrich), high glucose Dulbecco’s modified Eagle’s media (Cat. No. D6429, Sigma-Aldrich), or DMEM/F-12 HAM (Cat. No. D0547, Sigma-Aldrich) respectively, supplemented with 10% v/v FBS (Cat. No. F7524, Sigma-Aldrich), 1% v/v penicillin/streptomycin (Cat. No. P4333, Sigma-Aldrich) and 1% v/v Amphotericin B (Cat. No. 15290–026, Gibco). For experiments regarding extracellular pH, the pH of the media was adjusted to 6.0 by adding HCl. pH was measured after 24 h, and it was confirmed that it remained at pH 6.0. Cells were collected and seeded on glass coverslips or glass-bottom dishes coated with 10 μg/mL of fibronectin (Cat. No. RP43130, Invitrogen) in PBS (Cat. No. D8537, Sigma-Aldrich). Unless otherwise described, cells were cultured for 24 h before the pH was adjusted to pH 7.4 or 6.0 for an additional 24 h. Intracellular pH (pHi) was measured using Cell Meter Fluorimetric Intracellular pH Assay Kit (Cat. No. 21180, AAT Bioquest). For the Golgi localization experiment, cells were transfected with 2 μg of mEmerald-Golgi-7 (Cat. No. #54108, Addgene) cDNA using Lipofectamine 3000 (Cat. No. L3000001, Thermo Fisher Scientific) according to the manufacturer’s protocol.

The primary antibodies used in the experiments were anti-integrin β1 activated (Cat. No. MAB2079Z, Sigma-Aldrich), polyclonal anti-CRMP-2 (Cat. No. PA5–117919, ThermoFisher), polyclonal anti-phospho-CRMP-2 (Thr555) (Cat. No. PA5–143676, Thermo Fisher Scientific). The secondary antibodies and dyes used in the experiments were goat anti-rabbit IgG (H+L) Alexa-488 (Cat. No. A11034, Invitrogen, 1:400), anti-mouse IgG (H+L) Alexa-488 (Cat. No. A11029, Invitrogen, 1:200), and Alexa Fluor 546 Phalloidin (Cat. No. A22283, Invitrogen, 1:400), goat anti-rabbit IgG H&L (HRP) (Cat. No. ab6721, abcam, 1:3000). 24 h 10 μM Y-27632 (Cat. No. 688001, Sigma-Aldrich) treatment was used to inhibit ROCK-I and ROCK-II *in vitro*. A 30-minute treatment with 1 unit/ml CN01 (Cat. No. CN01, Cytoskeleton) and a 2 h treatment with 2 μg/mL CT04 (Cat. No. CT04, Cytoskeleton) were used in serum free media to activate or inhibit RhoA, respectively. Cell surface β1 and β3 integrins were blocked by pretreating the cell suspension for 10 min with 10 μg/mL of anti-β1 integrin (Cat. No. 552828, BD Biosciences) or 10 μg/mL of anti-αVβ3 integrin (Cat. No. MAB1976Z, Millipore) blocking antibodies, respectively, prior to seeding and providing 10 μg/mL of antibodies during the course of the experiment.

### 4.3 Microtubule Dynamics

For total internal reflection fluorescence (TIRF) microscopy, cells were cultured in glass-bottom Petri dishes to ensure high resolution and signal-to-noise. Cells were transfected with 1.5 μg EB1-RFP (Cat. No. 39323, Addgene) using Lipofectamine 3000 (Cat. No. L3000001, Thermo Fisher Scientific) transfection reagent 72 h before analysis. The pH of the medium was adjusted (pH 7.4 or 6.0) 24 h before analysis. Prior to measurements, the cell culture medium was changed to clear cell medium with adjusted pH to reduce autofluorescence from the cell medium.

TIRF images of transfected cells were obtained with an inverted microscope (Eclipse Ti; Nikon) operating in TIRF mode, under ambient temperature conditions of 37 °C. Time-lapse TIRF imaging was performed with a 63X oil immersion objective (1.49 NA, Nikon), a 488 nm diode laser for excitation, coupled with a 580 nm emission filter. Time-lapse images were recorded at 2 Hz using a sCMOS camera (Neo, Andor) combined with the NIS Elements (Nikon) control software. To minimize drift in the focus across time and multiple regions, the perfect focus system (Nikon) was used to maintain axial focus.

TIRF image sequences were analyzed in ImageJ, using the bleaching correction plugin to allow visualization of +TIP EB1 comets throughout the sequence. EB1 comet traces were tracked using TrackMate plugin^68^ from formation to disappearance from the focal plane (catastrophe). Track length and tracking time, representing microtubule length and polymerization duration, respectively, were obtained from the TrackMate traces and analyzed using Prism (version 9, GraphPad ).

### 4.4 Fluorescence Recovery after Photobleaching (FRAP) Analysis

FRAP analysis of microtubule dynamics was performed on glass-bottom Petri dishes (Mattek) coated with 10 μg/mL of fibronectin (Cat. No. RP43130, Invitrogen) in PBS (Cat. No. D8537, Sigma-Aldrich). Cells were transfected with 1.5 μg mCherry-α-Tubulin (Cat. No. 49149, Addgene) using Lipofectamine 3000 (Cat. No. L3000001, Thermo Fisher Scientific) 72 h prior to analysis. The extracellular pH was adjusted to the experimental conditions 24 h before imaging. For stabilization experiments, cells were treated with 10 nM taxol (Cat. No. P3456, Invitrogen) at the time of pH adjustment (24 h prior to imaging). Immediately prior to imaging, the culture medium was replaced with phenol red-free medium (DMEM, low glucose (Cat. No. D5921, Sigma-Aldrich)) with 0.584 g/L l-glutamine (Cat. No. G8540–25G, Sigma-Aldrich), supplemented with 10% v/v FBS (Cat. No. F7524, Sigma-Aldrich), 1% v/v penicillin/streptomycin (Cat. No. P4333, Sigma-Aldrich), and 1% v/v Amphotericin B (Cat. No. 15290–026, Gibco) adjusted to the specified experimental pH levels to minimize background fluorescence during data acquisition, with or without taxol.

Confocal photobleaching was conducted using an inverted microscope (Ti Eclipse, C2-SHS C2si Ready Scanner, Ti-TIRF-E Motorized TIRF Illuminator, CFI Plan Apo TIRF 60× NA 1.49 oil objective; Nikon). Five reference confocal images were acquired at 1 s intervals prior to bleaching. Specified regions of interest (ROIs) were bleached using a confocal laser at 8 mW. Following bleaching, images were captured at 1 s intervals for 120 s to monitor the fluorescence recovery kinetics.

Images were analyzed using ImageJ/FIJI to measure the mean gray value for each bleached ROI. Total fluorescence intensity was background-corrected and normalized to prebleach fluorescence intensity. Data analysis and curve fitting were performed using Prism (version 9, GraphPad). Fluorescence recovery curves were compared using the extra sum-of-squares *F*-test on the best-fit lines. The immobile fraction was calculated as $1-\frac{F_{\infty }-F_{0}}{F_{i}-F_{0}}$ , where *F*_∞_ is the fluorescence intensity at the end of the recovery phase (plateau), *F*_*i*_ is the prebleach fluorescence intensity, and *F*_0_ is the fluorescence intensity immediately postbleach. Error bars represent the standard error for each plateau. The half-time of recovery $\left(t_{1/2}\right)$ was derived from the nonlinear regression fits as the time required to reach 50% of the plateau intensity and is presented as the mean ± SEM for each condition.

### 4.5 Mechanical Stimulation

The impact of mechanical cues on β1 integrin activation was evaluated under both acidic (pH 6.0) and neutral (pH 7.4) culture conditions. For this purpose, pancreatic ductal adenocarcinoma Suit2 cells were cultured on soft (∼1 kPa) or stiff (∼10 kPa) elastomeric substrates. The soft and stiff substrates consisted of Dowsil CY52–276 mixed in 6:5 and 5:6 ratios, respectively (DowSil, Midland, MI). Prior to cell culture, the substrates were coated with 10 μg/mL fibronectin for 30 min. Cells were seeded at low confluency and cultured for 24 h as described in Section 4.2. After 24 h, the culture medium was adjusted to either pH 7.4 or pH 6.0, and cells were maintained for an additional 24 h before mechanical actuation. Moreover, 1 mM manganese chloride (MnCl_2_) was used as a positive control for β1 integrin activation, while 2 mM EDTA was used as a negative control by chelating extracellular calcium ions.

The mechanical actuation tests were conducted on cells cultured on the soft substrate. The stretching conditions were achieved by coupling the substrate to magneto-active domains following the methods described in previous work.^23^ Thus, external magnetic fields were generated using the NeoMag field generator (60Nd, Madrid, Spain), which induces control deformation within the substrate via the magnetostriction effect. Uniaxial strains of 30 ± 5% in magnitude were imposed for 15 min, followed by fixation with 4% paraformaldehyde. The effective transmission of these deformations from the substrate to the cells was validated by immediate cell deformation following mechanical actuation via imaging.

### 4.6 MD Simulations

In MD simulations, recent cryo-EM structures of α5β1 integrin in closed (7NXD) and open (7NWL) state (α5–res 1–603, β1–res 5–445) were used.^33^ p*K*_a_ values were predicted using PlayMolecule ProteinPrepare.^69^ All MD simulations were performed with Gromacs^70^ at Mahti supercomputer, CSC, Finland.

All MD simulations were performed using Amber14SB force field with SPC/E water model in 0.15 M KCl.^71^ The systems were energy-minimized and then equilibrated using harmonic position restraints on all heavy atoms of the protein. The temperature and pressure of the system were maintained at 300 K and 1 bar using V-rescale and C-rescale algorithms. An integration time step of 2 fs was used in all the simulations.

### 4.7 FRET Microscopy

Suit2 cells were cultured in glass-bottom Petri dishes, as described before. Cells were transfected with 1.5 μg pTriEx-RhoA-wt_mScarlet-i_SGFP2 (Cat. No. 85071, Addgene) using Lipofectamine 3000 (Cat. No. L3000001, Thermo Fisher Scientific) transfection reagent 72 h before analysis. The pH of the medium was adjusted (pH 7.4 or 6.0) 24 h before analysis. Prior to measurements, cell culture medium was changed to clear cell medium to reduce autofluorescence from the cell medium.

Confocal images of transfected cells were obtained with an inverted microscope (Stellaris 5; Leica) operating in FRET acceptor photobleaching mode, under ambient temperature conditions of 37 °C. Donor (SGFP) and acceptor (mScarlet) images were captured pre- and post-acceptor photobleaching using a 495 nm laser with a 512 nm emission filter, and a 570 nm laser with a 594 nm emission filter for the donor and acceptor, respectively. Images were analyzed using Leica LAS-X software for a quantification of acceptor photobleaching FRET efficiency. The ratio was obtained by subtracting the donor-emitted signal intensity before the photobleaching of the acceptor from its intensity after photobleaching the acceptor, normalized to the donor signal intensity post-photobleaching.

### 4.8 Western Blotting

The cell lysates were prepared with radioimmunoprecipitation assay (RIPA) buffer (Cat. No. 20–188, Millipore) containing protease and phosphatase inhibitors (Cat. No. 78440, Thermo Fisher Scientific). The protein concentration was quantified by DC protein assay (Cat. No. 500–0122, Bio-Rad) according to the manufacturer’s instructions. Samples were separated by a Tris-Glycine SDS–PAGE gel (Cat. No. XP00125BOX, Invitrogen) under reducing conditions and transferred to a nitrocellulose membrane (Cat. No. 10401196, GE Healthcare) then blocked with 5% bovine serum albumin (BSA, Cat. No. A8022, Sigma-Aldrich)–0.1% Tween-20 (Cat. No. P1379, Sigma-Aldrich) in TBS for phosphorylated CRMP, or blocked with 5% nonfat milk (Cat. No. M7409, Sigma-Aldrich) solution in 0.1% Tween-20 (Cat. No. P1379, Sigma-Aldrich) in TBS for total CRMP. All primary antibodies were prepared in the respective blocking solution and incubated overnight at 4 °C. The membrane was washed and incubated with horseradish peroxidase (HRP)-conjugated secondary antibodies in blocking solution for 1 h at room temperature. Finally, the membrane was washed and developed with HRP substrate (Cat. No. 34577, Thermo Fisher Scientific).

### 4.9 Immunofluorescence Staining

The cells were fixed with 4% w/v paraformaldehyde (Cat. No. P6148, Sigma-Aldrich) in PBS (Cat. No. D8537, Sigma-Aldrich) for 10 min, permeabilized with 0.5% w/v saponin (Cat. No. 47036, Sigma-Aldrich) in PBS, and then blocked with 1% w/v BSA (Cat. No. A8022, Sigma-Aldrich), 0.1% Tween-20 (Cat. No P6585, Sigma-Aldrich), and 22.52 mg/mL glycine (Cat. No. G8898, Sigma-Aldrich) in PBS for 30 min. After blocking, the cells were incubated with primary antibodies prepared in blocking solution overnight at 4 °C in a humid chamber. Then, the cells were washed in PBS and incubated with Alexa Fluor 488-conjugated secondary antibodies and phalloidin prepared in PBS for 1 h at room temperature. Finally, the coverslips were washed in PBS and mounted with ProLong Gold Antifade Mounting medium with DAPI (Cat. No. P36934, Invitrogen). Images in the mechanical actuation studies (Figure 3) were acquired at 40× magnification with a Leica DM6B upright microscope, equipped with ceramic objectives, and with Leica Application Suite X Software. Wide-field, immunofluorescent images in Figures 7, 8, S3, and S6 were acquired using a Nikon Ti-e Inverted Microscope (Ti Eclipse, CoolLED pE300 lamp, CFI Plan Fluor 40x NA 0.6 air objective; Nikon; Neo sCMOS camera; Andor) with NIS elements AR software.

### 4.10 Statistical Analysis

All statistical analyses were conducted with the Prism software (version 9, GraphPad). Data were collected from multiple repeats of different biological experiments to obtain the mean values and SEM displayed throughout. *p* values have been obtained using Mann–Whitney on unpaired samples, with parametric tests (*t* test) used for data with a normal distribution (following the Normality test). ANOVA with post hoc Tukey’s or Dunnett’s test was used to perform multiple comparison test on normally distributed data. Kruskal–Wallis test followed by post hoc Dunn’s multiple comparison test was used for multiple comparison tests with data that does not follow a normal distribution. Significance was set at *P* < 0.05 where graphs show significance through symbols (*0.01 < *P* < 0.05; **0.001 < *P* < 0.01; ***0.0001 < *P* < 0.001; ****P < 0.0001).

## Supplementary Material



## Data Availability

Data needed to evaluate the conclusions in the paper are present in the paper, and the raw materials data are available upon request.

## Notes

Additional experimental data, including intracellular pH measurements, microscopic images, and quantification of β1 integrin activation, CRMP-2, traction force generation, full Western blot membrane images, graphs of coordination distance of MIDAS and ADMIDAS simulations, and microtubule dynamics quantification tables (PDF)
