# Supplementary material for: Acidosis Regulates Microtubule Dynamics via the β1 Integrin/RhoA/CRMP‑2 Axis
Source: J Am Chem Soc. 2026 May 20;148(21):21279–95. doi: 10.1021/jacs.5c20041 (PMC13244432; doi:10.1021/jacs.5c20041)
Supplement: Supplementary file 1 [file ja5c20041_si_001.pdf]

Supporting Information:

Title: Acidosis regulates microtubule dynamics via the β1 integrin/RhoA/CRMP-2 axis

Authors: *Dariusz Lachowski, Ernesto Cortes, Vasyl Mykuliak, Miguel Fernandez-de la Torre, Ander Bastida Urkiza, Arrate Muñoz-Barrutia, Daniel Garcia-Gonzalez, Vesa Hytonen, Armando del Rio Hernandez****

Corresponding author: Armando del Rio Hernandez, email: a.del-rio-hernandez@imperial.ac.uk

This file includes:

Figures S1 to S7
Tables S1 to S3

Figure S1.

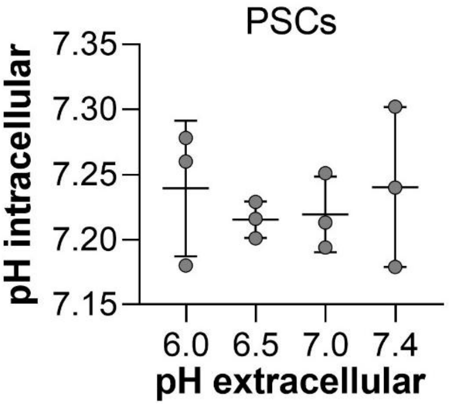

Extracellular acidosis does not affect intracellular pH. Intracellular pH measured by fluorometric intracellular pH assay in pancreatic stellate cells (PSC) cultured at different extracellular pH. No significant differences observed by one way ANOVA with Dunnett's multiple comparison post-hoc test.

Figure S2.

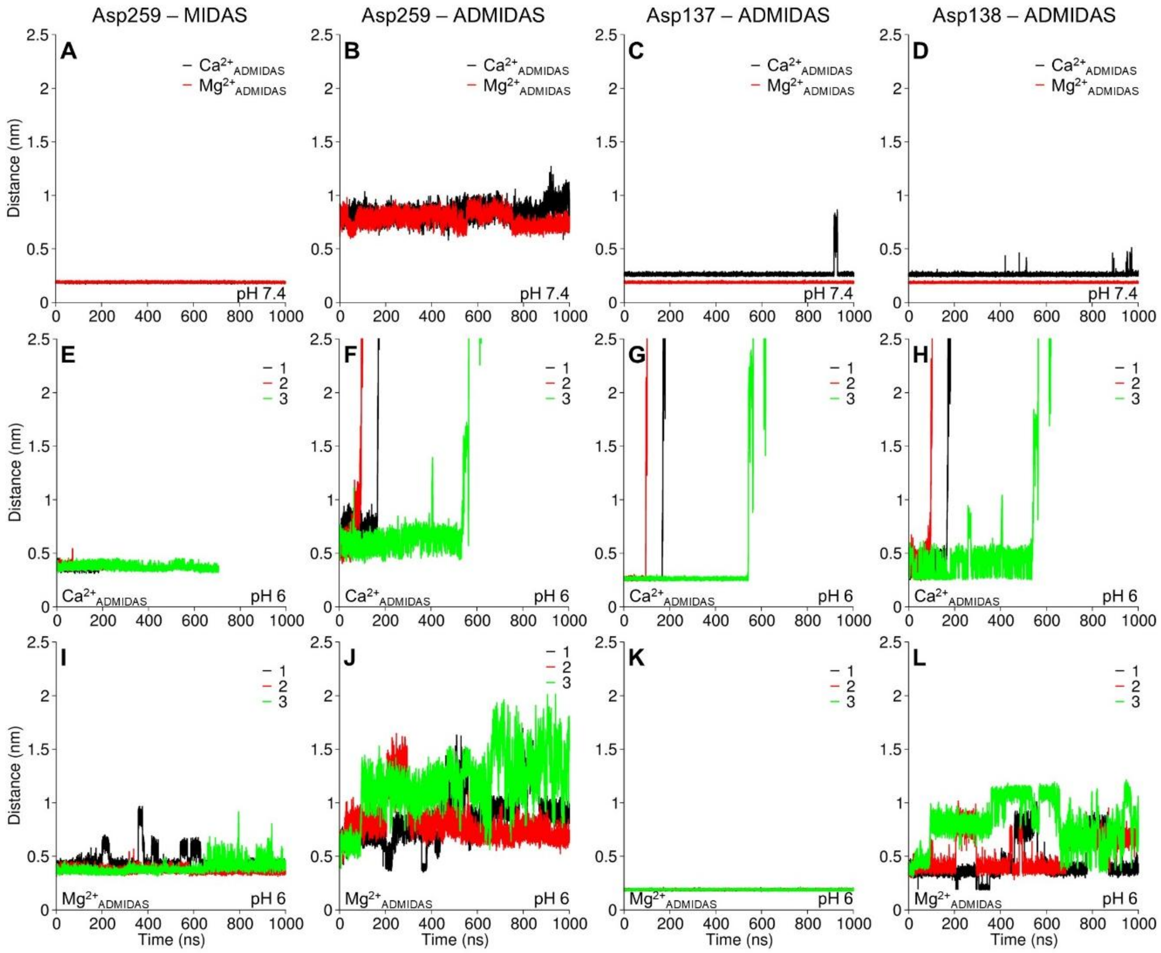

Coordination distance of MIDAS and ADMIDAS by Asp259, Asp137 and Asp138, with protonation state predicted for pH 7.4 and pH 6. Distance from the closest oxygen atom of **(A,E,I)** Asp259 to MIDAS, **(B,F,J)** Asp259 to ADMIDAS, **(C,G,K)** Asp137 to ADMIDAS, and **(D,H,L)** Asp138 to ADMIDAS. Two sets of simulations were performed, where MIDAS is always occupied by Mg^{2+}, while ADMIDAS and SyMBS with either Ca^{2+} or Mg^{2+}. One replica for each system was conducted using the protonation state predicted for **(A-D)** pH 7.4 and three replicas for pH 6 with **(E-H)** Ca^{2+} and **(I-L)** Mg^{2+} at the ADMIDAS and SyMBS. Asp138 is always protonated in our simulations at pH 6, and as result, **(F-H)** Ca^{2+} ion dissociates from the ADMIDAS during the 1 μs MD in all three replicas. **(E-H)** After Ca^{2+} dissociation, the MD simulation was terminated.

Figure S3.

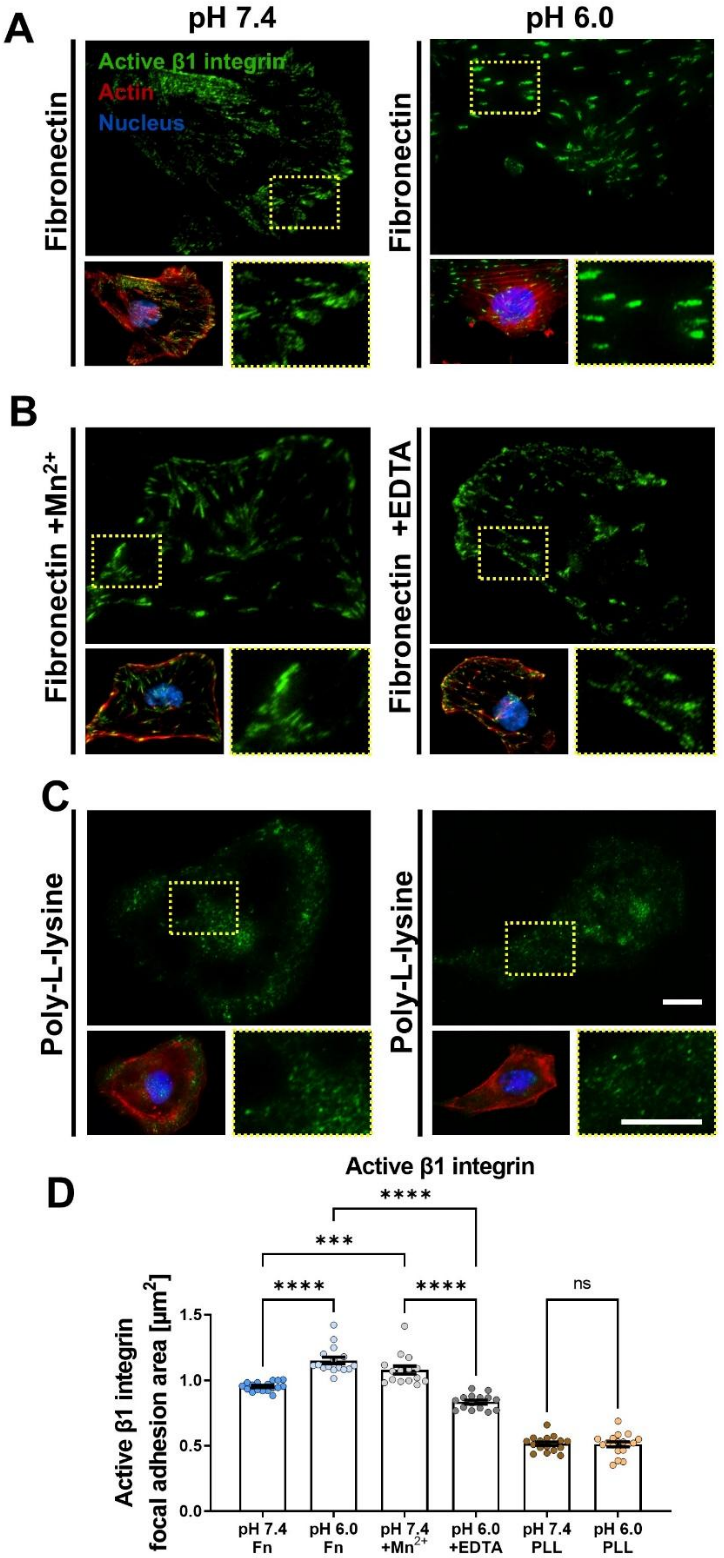

Extracellular acidosis increases β1 integrin activation. (A) Representative TIRF microscopy images of active β1 integrins in the basal plane of Suit2 cells cultured on fibronectin coated glass

in pH 7.4 media (pH 7.4 Fn), fibronectin coated glass in pH 6.0 media (pH 6.0 Fn), with 1 mM $MnCl_2$ treatment for 30 min in pH 7.4 (pH 7.4 +Mn^{2+}) and with 2 mM EDTA treatment for 30 min in pH 6.0 (pH 6.0 +EDTA), cultured on poly-L-lysine (PLL) coated glass in pH 7.4 media (pH 7.4 Poly-L-lysine), cultured on poly-L-lysine (PLL) coated glass in pH 6.0 media (pH 6.0 Poly-L-lysine). Scale bar 5 µm. **(B)** Mean active β1 integrin focal adhesion (FA) area. Histogram bars represent mean ± s.e.m., dots represent individual data points; n= 17, 16, 15, 15, 17, and 18 ROIs, 3 cells per ROI, for pH 7.4 Fn, pH 6.0 Fn, pH 7.4 + MN^{2+}, pH 6.0+ EDTA, pH 7.4 PLL, and pH 6.0 PLL respectively. Markers (*) denote significance between indicated groups by one way ANOVA followed by Dunnett's multiple comparison post-hoc test. n.s not significant, *** $0.0001<p<0.001$, **** $p<0.0001$.

Figure S4.

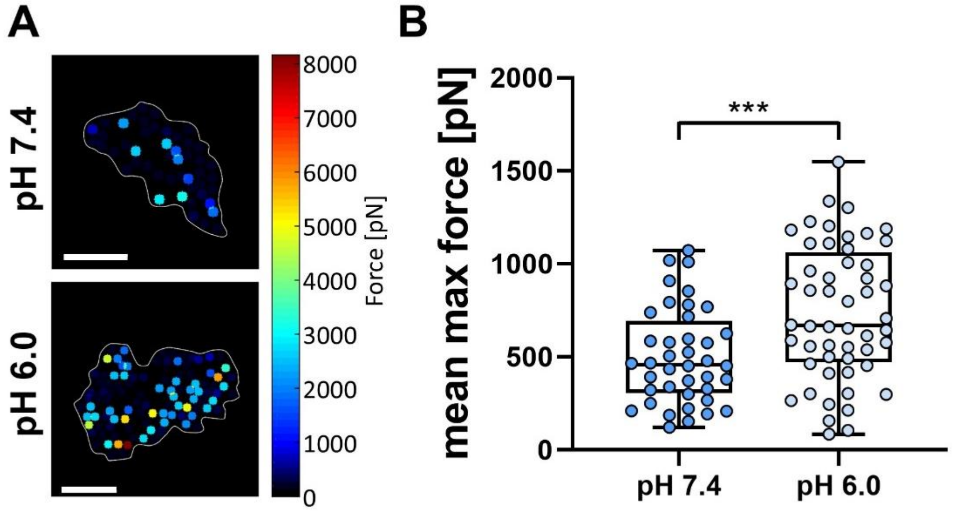

Extracellular acidosis increases traction force generation. (A) Heat maps of maximum traction force exerted by GE11 β1$^{+/+}$ mouse epithelioid cells on elastic pillar arrays. Scale bar 10 µm. **(B)** Mean maximum force generated by GE11 cells cultured based on (A). n= 40 and 52 cells for pH 7.4 and pH 6.0 respectively. Markers (*) denote significance by two-tailed unpaired t-test, *** p<0.001.

Figure S5.

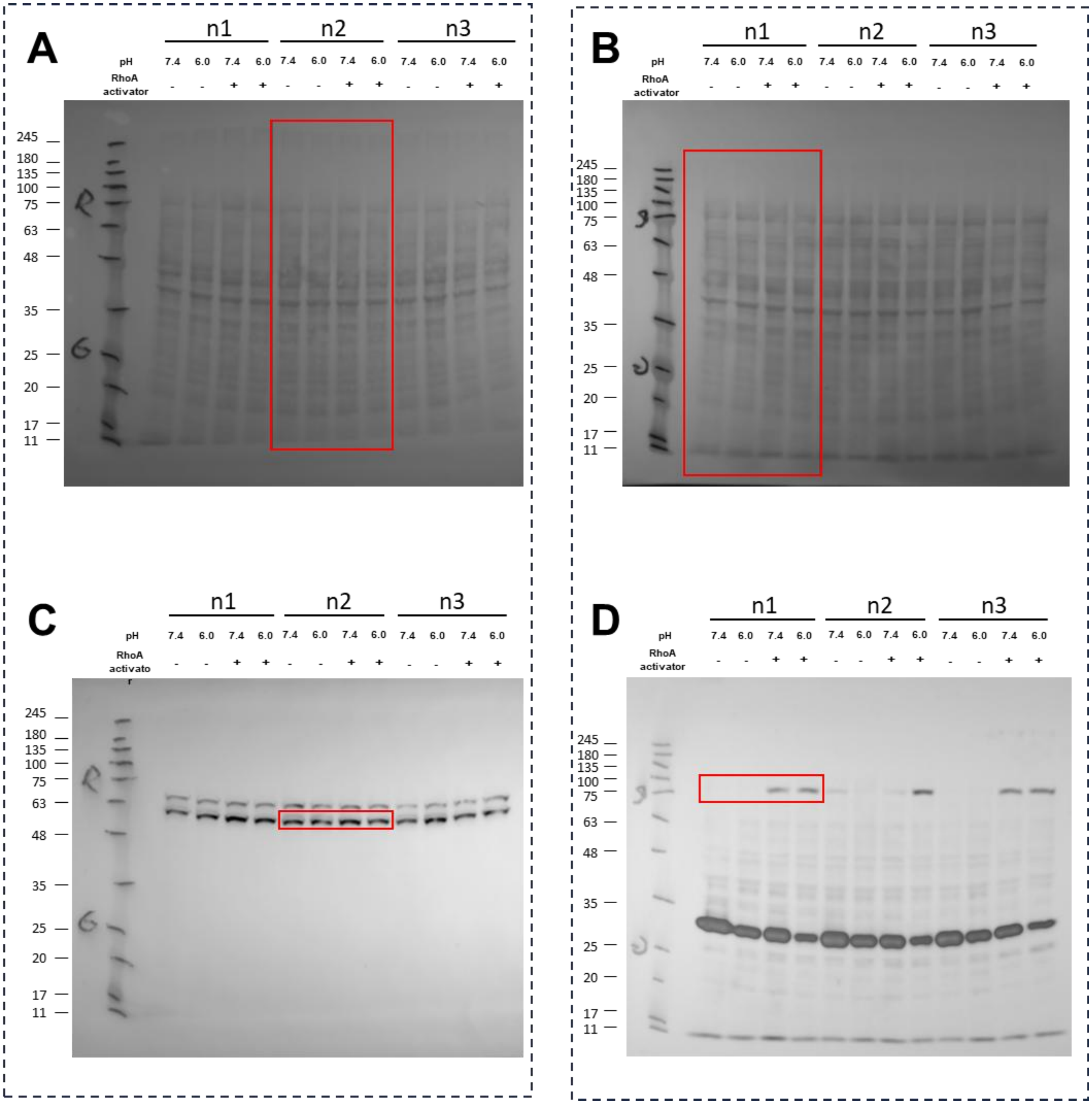

Full western blot membranes for CRMP-2 and pCRMP-2 (Thr555). **(A, B)** Ponceau Red total protein stain of membranes shown in **(C, D)** respectively. Full membranes of **(C)** total CRMP-2 and **(D)** pCRMP (Thr555). Images from the same membrane are indicated by a black dashed box, the cropped area presented in Figure 7 is indicated by a red box .

Figure S6.

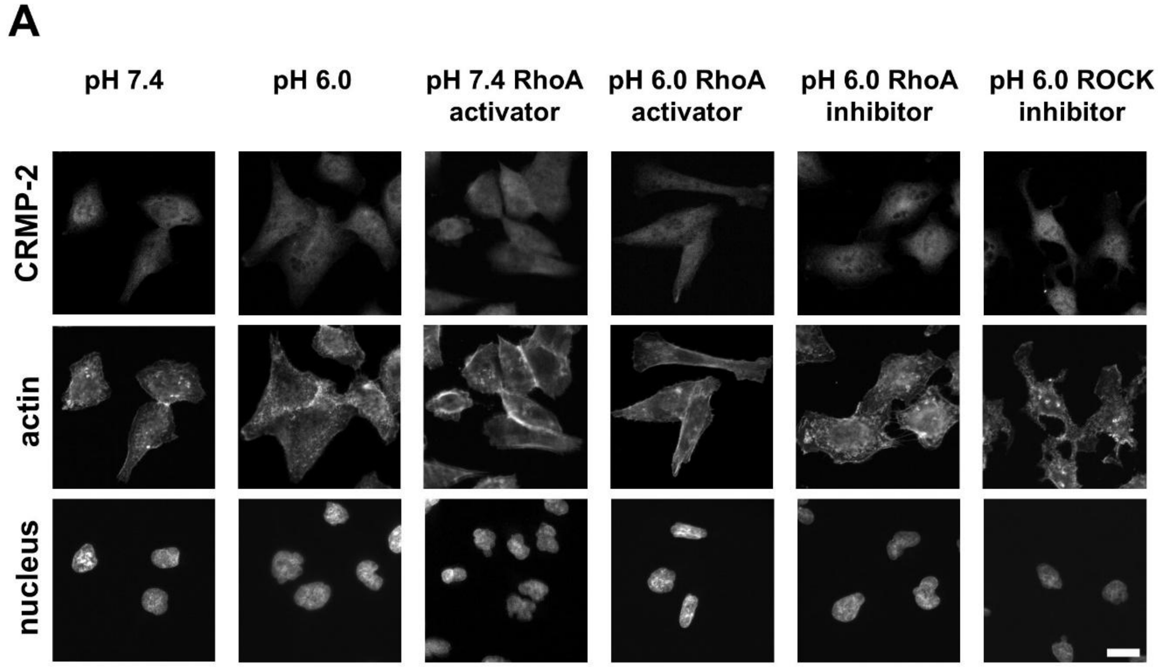

Acidosis does not affect the expression of CRMP-2 protein. (A) Widefield microscopy images for CRMP-2 in immunstained Suit2 cells at pH 7.4 , pH 6.0 , pH 7.4 with RhoA activator (CN01), pH 6.0 with RhoA activator (CN01), pH 6.0 with RhoA inhibitor (CT04) and pH 6.0 with ROCK inhibitor (y-27632). Scale bar 10 µm.

Figure S7.

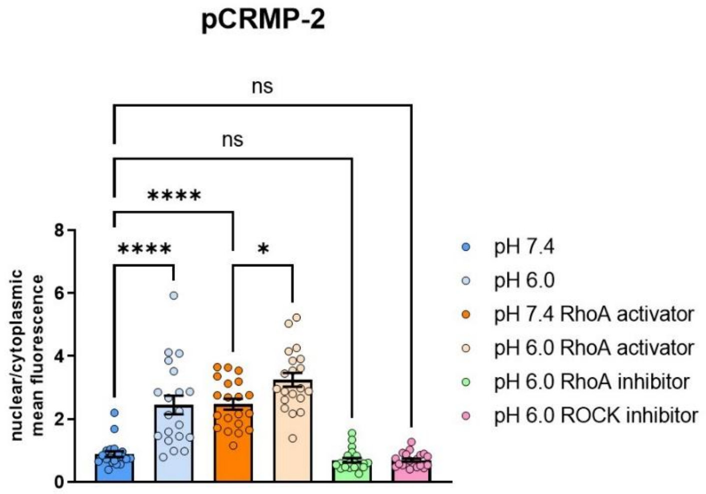

Acidosis increases pCRMP-2 nuclear location in a RhoA dependent manner. Mean pCRMP-2 (Thr555) nuclear to cytoplasmic ratio quantified using widefield images shown in Figure 6. Histogram bars represent mean ± s.e.m., dots represent individual cells; n= 20 for each bar. Markers (*) denote significance between indicated groups by one way ANOVA followed by Dunnett's multiple comparison post-hoc test. n.s not significant, * 0.01<p<0.05, **** p<0.0001.

Table S1.

Condition	n	Polymerisation time (s; mean ± SD)	Length (µm; mean ± SD)
pH 7.4	4461	8.148 ± 4.683	2.64 ± 1.33
pH 6.0	3864	5.763 ± 3.422	1.71 ± 1.39
pH 6.0 + β1 blocking	4761	7.267 ± 5.672	2.69 ± 2.38
pH 6.0 + β3 blocking	6971	5.637 ± 3.577	1.88 ± 1.31

Summary of microtubule dynamics by EB1 comet tracking in Suit2 PDAC cells. Data presented as mean ± SD.

Table S2.

Condition	n	Polymerisation time (s; mean ± SD)	Length (µm; mean ± SD)
pH 7.4 $\beta1^{+}$	2856	6.508 ± 4.972	1.53 ± 1.47
pH 6.0 $\beta1^{+}$	2561	4.499 ± 3.765	1.10 ± 1.22
pH 7.4 $\beta1^{-/-}$	3689	6.539 ± 5.554	1.52 ± 1.57
pH 6.0 $\beta1^{-/-}$	2206	6.598 ± 4.924	1.53 ± 1.55

Summary of microtubule dynamics by EB1 comet tracking in GE11 cells. Data presented as mean ± SD.

Table S3.

Residue	pKa, closed α5β1 headpiece	Charge at pH 7.4	Charge at pH 6
**Asp124**	6.5	–1	0
**Asp138**	7.2	–1	0
**Glu169**	7.6	0	0
**His263**	8.2	+1	+1
**Lys269**	6.0	0	0
**His282**	6.8	0	+1
**Asp295**	6.4	–1	0
**Glu432**	6.6	–1	0
**Asp228 (alpha5)**	6.4	–1	0

pKa values and protonation states of relevant amino acids.
